# Should Canada adopt managed access agreements in Canada for expensive drugs?

**DOI:** 10.1093/jlb/lsad014

**Published:** 2023-06-15

**Authors:** Melanie McPhail, Tania Bubela

**Affiliations:** Faculty of Health Sciences, Simon Fraser University, Burnaby V5A 1S6, Canada; Faculty of Health Sciences, Simon Fraser University, Burnaby V5A 1S6, Canada

**Keywords:** managed access agreement, drug reimbursement, health policy, drug regulation, expensive drugs, health technology assessment

## Abstract

Drugs are increasingly authorized based on less mature evidence, leaving payors faced with significant clinical and cost-effectiveness uncertainties. As a result, payors must often choose between reimbursing a drug that may not turn out to be cost-effective (or may even be unsafe) or delaying the reimbursement of a drug that is cost-effective and offers clinical benefit to patients. Novel reimbursement decision models and frameworks, such as managed access agreements (MAAs), may address this decision challenge. Here, we provide a comprehensive overview of the legal limitations, considerations, and implications for adopting MAAs in Canadian jurisdictions. We begin with an overview of current drug reimbursement processes in Canada, terminology and definitions of the different types of MAAs, and select international experiences with MAAs. We discuss the legal barriers to MAA governance frameworks, design and implementation considerations, and legal and policy implications of MAAs. Finally, we provide recommendations to guide policy development for implementing MAAs in Canada, based on existing literature, international experience, and our legal analysis. We conclude that legal and policy barriers likely prevent the adoption of a pan-Canadian MAA governance framework. More feasible is a quasi-federal or provincial approach, building on existing infrastructure.

## I. INTRODUCTION

Increased use of high-cost drugs has driven a rise in prescription drug expenditures. In Canada, expenditures rose 3.7 per cent from 2018/19 to 2019/20^1^, creating challenges for reimbursement decision-makers (payors) charged with the sustainability of public health systems. Standard cost-effectiveness assessments to determine whether, and under what conditions, drugs should be funded are not well suited for assessing high-cost new drugs, especially when they are authorized under accelerated regulatory pathways, based on less mature evidence. Increased uncertainty means that payors must often choose between reimbursing a drug that may not turn out to be cost-effective (or may even be unsafe), or delaying the reimbursement of a drug that is cost-effective and offers clinical benefit to patients. Novel reimbursement decision models and frameworks, such as managed access agreements (MAAs), may address this decision challenge. Payors in many countries have successfully adopted MAAs, however, uptake has been limited in Canada.

Here, we provide a comprehensive overview of the legal limitations, considerations, and implications for adopting MAAs in Canadian jurisdictions. In Part I, we provide an overview of current drug reimbursement processes in Canada. We then provide a brief overview of terminology and definitions of the different types of MAAs and an overview of select international experiences with MAAs, including the United Kingdom, Spain, Italy, the United States, and Israel. In Part II we discuss the legal barriers to MAA governance frameworks, design and implementation considerations, and legal and policy implications of MAAs. In Part III we describe the strengths and weaknesses of three potential MAA governance approaches (federal, quasi-federal, and provincial) that could be adopted in Canada. In Part IV, we provide recommendations to guide policy development for implementing MAAs in Canada, based on existing literature, international experience, and our legal analysis. We conclude that legal and policy barriers likely prevent the adoption of a pan-Canadian MAA governance framework. More feasible is a quasi-federal or provincial approach, building on existing infrastructure. Regardless of the approach adopted, many lessons may be learned from international experiences. MAAs can aid in managing rising drug expenditures and addressing clinical and cost-effectiveness uncertainties in the Canadian context.

## II. PART I

### II.A. Drug Reimbursement in Canada

After a new drug is authorized for sale by Health Canada, public and private drug insurance plans must decide whether, and under what circumstances, to reimburse the drug. Under existing processes, new therapies may be precluded from reimbursement because of difficulties in obtaining sufficient evidence to satisfy cost-effectiveness thresholds relied upon by reimbursement decision-makers.^2^ Additionally, reimbursement decisions have typically been static, made at one point in time during the drug’s lifecycle and therefore unable to adapt as new evidence emerges.^3^ As a result, there has been increasing interest, both in Canada and internationally, in adopting conditional reimbursement alternatives that grant temporary positive reimbursement status to a drug while confirmatory evidence is collected.

The Canadian Agency for Drugs and Technologies in Health (CADTH) is an independent, not-for-profit, quasi-federal organization responsible for providing reimbursement recommendations to participating drug plans. All the provinces and territories (with the exception of Quebec) participate in the CADTH Health Technology Assessment (HTA) process, in addition to the six federal drug plans.^4^ In Quebec, the Institut national d’excellence en santé et en services sociaux (INESSS) makes recommendations to the provincial Minister of Health and Social Services, who ultimately decides whether to reimburse a drug. Some provinces and territories also conduct their own HTA analyses to make decisions about whether drug products should be covered by public drug plans. Additionally, private drug plans develop their own formularies and reimbursement criteria, with many providing additional coverage for cancer medications.

Most, but not all, provinces have legislation giving the appropriate Minister the authority to add and remove drugs from public drug plan formularies for any reason, without notice.^5^ Most provinces differentiate between drugs that are funded unconditionally and drugs that require prior approval, but each province has different procedures for document submission, prescriber designation and exemption, and verification of previous prescriptions dispensed. Across provinces, there are differences in capabilities, coverage, and processes for reimbursing and accessing drugs.^6^

### II.B. Managed Access Agreements

#### II.B.1. Terminology

Though there is no one agreed upon definition of an MAA^7^ their purposes include: encouraging appropriate prescribing and utilization, improving access to medications, collecting additional evidence, and providing greater budget certainty.^8^ Different features of MAAs can be implemented depending on the purpose and desired outcome of each agreement. These features can be categorized as financial design features and clinical design features, although many agreements contain both. (Table 1).

**Table 1:** Overview of managed access agreement types

Type of agreement		Description
Financial	Leasing	Spread payments across utilization period
	Rebate/discount	Reduce listing price for all or some uses
	Refund	Payment refunded for ineffective treatment
	Price–volume agreement	Refund or discount total expenditure over pre-determined threshold(s)
	Utilization caps	Individual utilization above pre-determined threshold discounted or refunded
	First doses free	Payor does not pay for initial doses to identify responding patients
	Market share	Price adjusted based on total market share
	Price adjustment	Price adjusted based on utilization, outcomes, or evidence collected during
Clinical	Coverage with evidence development	Treatment temporarily reimbursed while additional evidence collected (either from patients treated via the agreement or more broadly)
	Conditional treatment continuation	Continued access to reimbursed treatment limited to patients who meet ongoing eligibility requirements
	Outcome guarantee	Reimbursement linked to individual patient response
	Process of care	Reimbursement tied to impact of treatment on clinical decision-making or practice patterns

Financial design features of MAAs include leasing, rebates or discounts, refunds, price–volume agreements, utilization caps, first doses free, market share, and price adjustments. Leasing agreements replace up-front payment for health technologies with a stream of payments spread over the expected duration of benefit from using the technology, subject to meeting predetermined outcomes.^9^ Rebates or discounts can be applied to listing prices automatically or tied to short- or long-term performance, where a drug manufacturer must reimburse the payor a predetermined portion of the purchase price based on individual outcomes or broader system usage. Rebates or discounts are simple mechanisms but require easy to measure outcomes and data sharing with drug manufacturers.^10^ MAAs can also require full refunds where specific outcomes are not achieved or maintained. Free treatment to a new patient may be provided in lieu of a refund. Similar to rebates, refunds require the ability to clearly define outcome metrics and processes for collecting refunds.^11^

Price–Volume Agreements (PVA) focus on controlling total financial expenditure by requiring the pharmaceutical company to refund total expenditure over the predetermined budget amount, issue partial refunds after the threshold is reached, or reduce prices in inverse proportion to sales volume.^12^ Similar to PVAs, utilization caps structure payment based on individual-level use, with the payor paying for utilization up to the predetermined threshold, and the manufacturer reimbursing or discounting utilization above the threshold.^13^ The opposite of utilization caps, under first doses free agreements the manufacturer provides a predetermined amount of first doses free to help identify responders.^14^ Finally, market share agreements or price adjustments may be used to adjust the price at set points. Market share agreements include terms to adjust the price of a drug based on total market share. This type of agreement is most appropriate where there are numerous available treatments.^15^ Alternatively, in price adjustment agreements the price of the drug is adjusted based on pre-determined use or outcomes following the completion of the MAA and based on an assessment of the evidence collected during the term of the agreement.^16^

Instead of, or in addition to financial design features, MAAs may include clinical design features of MAAs, such as coverage with evidence development (CED), conditional treatment continuation, outcome guarantee, or process of care. In CED agreements, a drug is reimbursed while additional evidence is collected to reduce the payor’s uncertainty about clinical and/or cost-effectiveness of the health technology. Access to health technologies under CED agreements can be ‘only in research’, where patients can only access the reimbursed health technology by participating in a research program and contributing their health data, or ‘only with research’, where patients can access the reimbursed health technology with or without contributing their data.^17^ Conditional treatment continuation links continued access to reimbursed treatment to patients meeting predetermined thresholds demonstrating the drug’s clinical effectiveness.^18^ Outcome guarantees are usually linked to rebates, refunds, discounts, or price adjustments. They require collecting individual patient data to assess the performance of the medication and linking reimbursement to the data collected based on a predetermined formula.^19^ Lastly, process of care agreements tie reimbursement to the impact of the introduction and utilization of the health technology on clinical decision-making or practice patterns.^20^

#### II.B.2. MAAs in Canada

Most MAAs in Canada were concluded prior to the establishment of the pan-Canadian Pharmaceutical Alliance^21^ to conduct negotiations on behalf of provincial/territorial drug plans.^22^ The pCPA’s mandate is to enhance patient access to clinically relevant and cost-effective drug treatment options by conducting collective, expert-informed negotiations for drugs. All brand name drugs coming forward for funding through the national review processes are assessed by the pCPA before negotiations are considered.^23^ If the pCPA pursues negotiation, the negotiation will either result in mutually agreed upon terms and a fully executed Letter of Intent, or a close letter is issued. If negotiations are successful, the letter of intent sets out the effective price that public payors are willing to pay and any other rights and restrictions, including rebates, caps, and termination rights. Individual plans then use the letter of intent to finalize negotiations and execute a product listing agreement (PLA). Letters of intent are non-binding and serve as a basis for each jurisdiction to sign individual PLAs.^24^ The terms of letters of intent are confidential, and there is currently no mechanism to determine how closely final PLAs align with pCPA negotiated terms.

The use of PLAs within and outside the pCPA process is inconsistent across Canada; while some provinces use PLAs almost exclusively (Ontario and Manitoba), others never use them (Quebec and Newfoundland and Labrador). Alberta has perhaps the most well-developed PLA program, including a policy that stipulates comprehensive parameters for establishing and executing four types of PLAs: (i) price–volume agreements; (ii) health research capacity agreements; (iii) utilization management agreements; and (iv) CED. The Ministry of Health issues a request for PLAs, following which drug manufacturers can submit a proposal for a PLA. Other provinces have indicated a desire to implement PLA policies similar to Alberta’s.^25^

British Columbia, on the other hand, does not have a detailed, publicly available process for implementing PLAs, but one study found that 7/14 reviewed drugs used PLAs.^26^ Similarly, Ontario does not have an official mechanism for negotiating and implementing PLAs. However, Ontario does have a policy that allows the implementation of CED agreements under the Evidence Building Program (EBP). As of Dec. 2020, only two drugs, Herceptin and Oxaliplatin are funded via the EBP, even though the policy has been in place since 2011.^27^ Though PLAs are legal in Quebec, they are not used. Instead, Quebec has a lowest price rule, which prohibits drugs from being priced higher than the same drug in any other Canadian jurisdiction.^28^ The increasing use of PLAs, which often mask the true price of the drug, has made the implementation of this rule challenging.

PLA use in Canada has grown, attributed to increasing CDR recommendations that include price reductions, increasing payor uncertainty about the value of drugs, and increasing pressure to provide access to medicines within budget.^29^ The most common type of PLAs used by the provinces is financial agreements that avoid the administrative issues associated with clinical MAAs. Simple discounts or rebates, utilization caps, and PVAs are the most common. Innovative MAAs, such as indication-specific pricing, conditional treatment continuation, and CEDs are reported in only a handful of cases.^30^

Though the contents of PLAs and MAAs are often confidential, a 2010 review identified three risk-sharing agreements in Canada. First, Sandoz, a division of Norvartis, agreed to reimburse hospitals and government drug plans if patients with treatment resistant schizophrenia discontinued clozapine within six months, to address acquisition cost concerns compared to typical anti psychotics. In another agreement, Merck-Frost offered to reimburse provincial governments the full cost of treatment if patients prescribed finasteride subsequently required surgery for benign prostatic hyperplasia after one full year of medical therapy. Third, Sanofi-Aventis agreed to reimburse the cost of docetaxel to provinces if an agreed upon responder level was not reached in patients with cancer due to concerns about its efficacy and cost. The program lasted 6 months, serving as an interim measure prior to formal reimbursement.^31^

More recently, Toumi and Jaroslawski conducted a global analysis of the use of MAAs, in which they identified three distinct MAAs not previously identified in the 2010 review. A 2005, CED agreement with Genzyme and Shire for enzyme replacement therapies in Fabry disease was entered into to address negative public reception of a negative reimbursement decision and collect further data in a formal post-market stud, which went on to support the final positive reimbursement decision. Second, in 2017, pCPA entered into an agreement with three hepatitis C drug manufacturers to secure a therapeutic class discount. Third, following a negative reimbursement recommendation from CADTH for teduglutide in short bowel syndrome, Shire entered into a P4P agreement with pCPA, which included a patient support program and payment only for responders.^32^

There remains great stakeholder interest in and need for MAAs in Canada, but several barriers and gaps have been identified through stakeholder interviews. While willingness to use MAAs is high, systems readiness is low. Lack of understanding and knowledge regarding design and implementation of MAAs, concerns about transparency and sharing experience, and stakeholder alignment have been identified as key factors supporting the future success of MAAs in Canada. The following section provides a comprehensive overview of potential and actual legal, regulatory, and pragmatic barriers and enablers for the continued development of MAAs in Canada.

### II.C. International Experience with MAAs

Though Canada has unique geo-political features that limit the transfer of models from other jurisdictions, there are lessons that can be learned from international experiences with the design and implementation of MAAs. We review examples from the United Kingdom (UK), Spain, Italy, the United States, and Israel.

#### II.C.1. United Kingdom

In the UK, the National Health Service (NHS) funds most drugs and health services for patients. As the gatekeeper to reimbursement, the National Institute for Health and Care Excellence (NICE) has the authority to make recommendations, including that health bodies provide funding within a specified period to make health technologies available to patients. Health bodies are required to comply with positive recommendations; as a result, the NHS is legally required to fund drugs recommended by NICE, but a negative recommendation does not preclude reimbursement.^33^

NICE conducts a health technology appraisal for all new drugs launched in the UK. The three pathways for NICE to recommend alternative reimbursement mechanisms are: the Cancer Drugs Fund (CDF), Patient Access Schemes (PAS), and MAAs. The CDF allows patients to access innovative new cancer drugs based on draft recommendations issued prior to receiving the license.^34^ If a drug is determined to have potential to satisfy the criteria for recommendation, but there is significant unresolved clinical uncertainty, which requires more investigation, the drug will be made available under the CDF, while additional real-world evidence is gathered to resolve the uncertainties.^35^ Drugs reimbursed under the CDF are reviewed at a predetermined period, after which a final recommendation is made. A review of the CDF from 2010 to 2015 found that the fund spent £1.3 billion to pay for cancer treatments that had failed to gain NICE approval. Only one-third of these treatments had demonstrated an effect on overall survival at a median of 3.1 months. The authors of this study concluded that the CDF failed to deliver meaningful value to patients or society.^36^ A new fund started in July 2016, which has been reported as being successful at driving down prices.^37^

When a product does not meet NICE’s cost-effectiveness criteria, NICE may still make a positive recommendation if the drug supplier agrees to a PAS, a formal pricing agreement between a supplier and the NHS that makes a product more affordable by way of a price discount, rebate, free-stock, or outcome-based pricing. The *Pharmaceutical Price Regulation Scheme (2014)* (the *PPRS*) states that PAS are intended to be an exception, rather than a standard reimbursement pathway.^38^ There are two categories of PAS: simple discount schemes and complex schemes. Simple discount schemes are the preferred model as they impose no ongoing additional burden. Complex schemes could include rebates, free doses, dose capping, and outcomes-based schemes. Experience to date with complex schemes demonstrated the high burden both on industry and the NHS, further supporting their use only in exceptional circumstances. An important feature of a PAS is a built-in review date to assess the performance of a PAS and identify any appropriate changes, if necessary. PAS are expected to remain in place for the lifetime of the guidance.^39^

Similarly, where the clinical data are uncertain, NICE may recommend a product be reimbursed subject to an MAA, which enable NHS patients to access treatment while real-world data is collected for a reappraisal. All drugs recommended under the CDF utilize MAAs, including a Data Collection Agreement, which sets out the outcomes that need to be collected, and a CDF Commercial Agreement to determine the cost of the drug during the managed access period. The data collection arrangement will vary from drug to drug depending on the area(s) of clinical uncertainty described by the NICE Technology Appraisal Committee. The timeframe of interim access and data collection is intended to be as short as possible, up to two years, but is determined on a case-by-case basis.^40^

While a drug is in the CDF, data are collected per the MAA. All chemotherapy providers in England are required to have an electronic prescribing system in place. Clinicians or hospitals must submit a request for funding for drug/indications on the CDF (which is updated and circulated). A requirement of funding is the submission of all required clinical and financial treatment data. Once sufficient data have been collected to answer the original uncertainty, NICE will schedule a re-appraisal. The outcome of the re-appraisal will either be a positive recommendation for routine commission or a negative routine commissioning recommendation. If the latter, funding is only available in exceptional circumstances.^41^

The manufacturer of the technology under review, patient groups, or clinician organizations who have participated in the assessment may appeal the outcome of an NICE assessment to the NICE Appeal Panel. The three possible grounds for appeal mirror the grounds of judicial review in English courts: failure to act fairly; the recommendation is unreasonable in light of the evidence submitted; and/or NICE acted unlawfully or exceeded its legal powers. If an appeal to NICE’s Appeal Panel is unsuccessful, a party may seek Judicial Review in the High Court.^42^ For example, a recent decision from the England and Wales Court of Appeal (Civil Division) found that a decision to exclude a patient from eligibility to treatment under an MAA was unlawful and irrational.^43^ The plaintiff was excluded from accessing nusinersen, treatment for Type 3 Spinal Muscular Atrophy because she did not meet criteria required by the MAA. Specifically, the plaintiff’s doctors determined that she did not meet the 5 Steps Criterion, which requires the patient was able to walk five steps unaided in the twelve months prior to initiating treatment. However, the plaintiff had not previously been assessed on this metric as it was not part of routine clinical practice. The plaintiff’s family submitted evidence that she did meet the criteria, but the NHS concluded that there was insufficient clinical evidence to change the initial decision. The plaintiff’s mother, as a litigation friend, initiated judicial review of the decision not to prescribe nusinersen. The Judge dismissed the application, concluding that assessing eligibility criteria required expert clinical judgement, and was not a simple question of fact. Sophie’s mother appealed the decision to the England and Wales Court of Appeal.^44^

In the Appeal, the Court held that the judge erred in categorizing the decision as one of expert clinical judgement, reasoning that a substantial part of determining whether the patient met the criteria was a simple question of fact. Instead, the court held that a conventional judicial review approach with deference to clinical judgement was appropriate. As a result, the Court found that the decision of Sophie’s doctor to not prescribe her nusinersen was unlawful and irrational, and the NHS was ordered to reconsider the decision. In June 2021, NICE released an updated MAA, removing the 5 step criteria.^45^

Several lessons can be gleaned from the UK’s experience with MAAs. First, in the UK health bodies are mandated to fund drugs that are given a positive recommendation by NICE. No such requirement exists for CADTH recommendations. As a result, provincial funding decisions can vary across the country. Though it is likely not possible within Canada’s existing constitutional structure to authorize CADTH to make binding recommendations, this barrier to adopting more consistent reimbursement decisions in Canada will be an important factor in designing and implementing MAAs. Second, the UK has integrated data and administrative systems that allow for MAAs to be utilized without creating new data and administrative infrastructure. Such systems do not exist across Canada, or even consistently within individual provinces to support the implementation of MAAs. The UK’s integrated systems minimize the burden of verifying eligibility criteria and reimbursing individual institutions. In contrast, administrative health data systems in Canada need modernizing and continue to rely on complex and inefficient data sharing arrangements. Third, the *Basma* case demonstrates that MAAs need to be designed with consideration of existing clinical pathways. Eligibility criteria should be assessed to ensure that their application will not prevent patient access because included metrics are not routinely assessed in clinical practice. Fourth, appeal processes are important features of MAAs. If a patient, physician, or drug manufacturer disagrees with a decision made within a MAA, clear processes for appeal are necessary to ensure that MAAs are applied fairly. Fifth, NICE uses a multi-disciplinary approach to design, implement, and assess MAAs. Patient groups, the drug manufacturer, clinicians, and NICE are all involved in designing the terms of the agreement and reviewing interim and final data analyses. Involving multiple stakeholders helps ensure that varying interests and perspectives are considered and represented in the agreement and can mitigate the impact of unintended consequences. Finally, under at least some MAAs in the UK, data collected under the MAA are owned by the drug manufacturer, despite being collected within a publicly funded health care system. Though there are agreements in place to ensure data sharing with all signatories to the agreement, manufacturer-owned data may raise concerns for data security and reliability.

#### II.C.2. Spain

Spain is a decentralized, unitary country with 17 autonomous communities and 2 autonomous cities. As a result, each autonomy has full sovereignty, unlike Canadian provinces and territories, which share jurisdiction with the federal government. Drug coverage is the responsibility of the federal government in Spain, while each autonomous community is responsible for ensuring public access to national health services, included drugs covered by the federal government.^46^

The Catalonia region has the most experience to date with risk-sharing agreements (RSAs). In Catalonia, the Catalan Health Service (CatSalut) is responsible for managing health services, including access to drugs. CatSalut has used flexible reimbursement systems for medicines linked to results since 2011. For the first five years, the program focused on pilot projects at the institutional level. These were led by the hospitals and self-managed in terms of clinical and financial assessment and administrative tasks. In 2016, CatSalut took the lead, introducing a centralized system. RSAs were implemented by a single contract between market authorization holders and CatSalut. Participation in financial arrangements was automatic and mandatory, but participation in performance-based agreements was voluntary and required an adhesion contract between individual hospitals and the market authorization holder. In 2020, CatSalut made the participation in performance-based RSAs automatic and mandatory, eliminating the need for adhesion contracts.^47^

The majority of RSAs in Spain are for oncology, rare disease, and neurological indications. Various MAA mechanisms are used, including discounts, budget capping, and PVAs. Generally, Spain relies on short-term RSAs, with a mean duration of 24 months and clinical evaluation of 2–24 weeks. Notably, under CatSalut’s RSA program, even if patients do not meet the inclusion criteria for the RSA, their treatment is fully reimbursed.^48^ Though formal evaluation of RSAs is not routinely conducted, there are other oversight mechanisms in place. For example, each RSA has its own follow-up committee with annual meetings to share data and experience.^49^ There have also been a few external evaluations of individual RSAs in Spain. For example, one evaluation of the RSA for gefitinib to treat non-small cell lung cancer found that the RSA resulted in average savings of €800 per patient and €36,000 in total savings for the health system.^50^ Another evaluation identified 15 RSAs in Spain, for which €2.4 million was refunded, equivalent to 3.9 per cent of total expenditure. Additionally, none of the medications funded under budget capping schemes reached their threshold of use, suggesting they were effective in limiting overuse. However, of the five RSAs that concluded during the study period, only two had uncertainties sufficiently addressed; the remainder was terminated, or evaluation was hindered by insufficient data.^51^ Despite the regional approach typically taken in Spain, a few national level RSAs have operated in Spain. Biogen has negotiated two agreements with the Spanish Ministry of Health; one in 2013 for Fampyra to treat multiple sclerosis, and a second in 2018 for Spinraza to treat spinal muscular atrophy.^52^

Reviewing Spain’s experience with RSAs demonstrates the importance of ensuring the sufficient provision of resources (financial, technical, administrative, and human) to implement and monitor RSAs. Catalonia has experienced some challenges and additional costs that result from additional staff time, technology needs, and diagnostics; however, direct costs can be difficult to identify. Specifically, access to suitable databases is the ‘first and most important step…to securing a successful RSA implementation.’^53^ Catalonia’s approach demonstrates that regional data systems can, in some situations, be sufficient for RSAs. Catalonia’s population is similar to many mid-size provinces in Canada; however, RSAs for rarer diseases may not be feasible in smaller data systems. Second, Catalonia’s RSA guideline was largely based on academic input, which undervalued the impact of real-world limitations and challenged existing methodologies for data analysis. Any MAA program adopted in Canada will need to be developed after accounting for methodological limitations presented by data and clinical systems. Third, Catalonia successfully introduced RSAs through an iterative implementation process, starting with individual hospital pilot programs and eventually mandated participation across all institutions. This approach may be advisable in Canada, while more integrated data and admirative systems are developed and implemented. Small pilot projects at individual hospitals or centers will permit Canadian-specific legal and practical factors to be identified, and solutions tested prior to widespread adoption. Implementing agreements on a voluntary basis for an initial period is also beneficial to permit institutions sufficient time to plan and prepare for any necessary updates or institution-specific factors that need to be addressed. Lastly, comprehensive evaluations are not routinely completed of RSAs in Spain. This makes it difficult to understand the actual clinical, financial, and administrative costs and gains realized by RSAs and represents a lost opportunity to identify areas for improvement and continued justification for use of RSAs.

#### II.C.3. Italy

Italy is one of the most active MAA users in Europe. The Italian Medicines Agency (AIFA) is responsible for regulatory authorization, pricing, reimbursement, and health technology assessment.^54^ MAAs have become standardized within Italy’s drug regulatory process. AIFA relies heavily on comprehensive data collection and online patient monitoring registries that permit continuous evaluation of real-world drug use that enable analysis for the purposes of MAAs.^55^

AIFA utilizes various types of registries, including: drug product monitoring registries, therapeutic indication monitoring registries, and therapeutic plan registries. AIFA registries collect administrative data from hospitals, pharmacies, regional and district health services, and drug manufacturers. The registry platform supports automatic linkage of records across registries and utilizes a standardized system for adopting and designing registries. AIFA uses both patient-level and population-level approaches; including different types of MAAs: appropriate prescribing agreements, outcome agreements (risk sharing and payment by results), and financial agreements (cost-sharing and capping).^56^ Though there is no formal evaluation mechanism, some independent reviews of Italy’s experience with MAAs have been conducted. One analysis identified 283 indication-based registries up to the end of 2019; of those, 64 per cent were for appropriateness MAAs^57^, 12 per cent were appropriateness plus financial arrangements, and 21 per cent were appropriateness plus outcome agreements.^58^ It has been estimated that MAAs saved the Italian health system €531.8 million in 2017, while the costs of managing registries is estimated at approximately €1 million.^59^

Independent analyses have identified a few challenges. One analysis in Italy found that enforcing refunds has been problematic; only 67 per cent of refunds from certain agreements were recovered because of challenges with refund notifications and lack of incentive for institutions to participate.^60^ Similarly, data collected by healthcare professionals were often insufficient because of subtotal compliance with registry procedures.^61^ This may be due to a lack of incentives for healthcare professionals to complete the necessary data entries and claim paperwork as well as the complexity of using heterogeneous, unstandardized registries.^62^ Another review suggested that there were insufficient measures in place to ensure unbiased selection of patients, potentially undermining the data collected and subsequent decisions.^63^

One of the strengths of Italy’s approach is the use of multiple tools to achieve specific ends. Italy uses different types of registries and agreements depending on the context. While this approach permits significant flexibility to collect tailored evidence and address different forms of uncertainties, it can also introduce inefficiencies by requiring additional infrastructure, human resources due to lack of standardization and longer learning curve. Finding an appropriate balance between flexibility and certainty to maximize efficiency, compliance, and usability is important to facilitate implementation and justify the added burden associated with data collection.

Additionally, Italy is one of the only countries that consistently uses indication-based pricing.^64^ Such an approach will be increasingly necessary as more precision oncology drugs come to market, which are often authorized and funded for use in multiple indications, with differing cost-effectiveness per indication. In many jurisdictions, including Canada, current systems do not enable indication-based pricing. Another unique feature in Italy’s approach is the use of patient registries to complement existing health administrative data systems. Such an approach may be beneficial in Canada, where the use of administrative health data is hindered by structural and legal realities. Patient registries can be set up and tailored to context, including drug, disease, and patient population. However, experience in Italy indicates that a lack of standardization has imposed additional burden on stakeholders and systems, resulting in compliance issues. Standardizing agreements and procedures as much as possible, while retaining flexibility to adapt to specific scenarios, may mitigate these challenges, especially if accompanied by widespread stakeholder education and appropriate incentives for compliance. Italy’s experience may also provide some guidance on funding arrangements. Patient registries are funded by industry but governed by AIFA.^65^ Such an approach can help mitigate the costs borne by the public health system to collect post-market evidence.

#### II.C.4. United States

Though the predominance of private health insurance in the United States limits relevance to Canada from a governance perspective, private and public drug insurers have experience implementing a variety of MAAs. Reports as early as the 1990s document use of MAAs in the US; for example, Merck promised to refund prescription costs if simvastatin plus diet did not help lower cholesterol to target levels. There are numerous examples of similar agreements between manufacturers and private health insurers, largely utilizing rebates for failed treatment.^66^ Due to the confidential nature of such agreements, there is limited information available and they are currently negotiated in an ad hoc fashion with no overarching governance framework.

In the public sphere, the Centers for Medicare and Medicaid Services (CMS) has utilized CED since 2006. After a drug is authorized by the US FDA, CMS assesses whether the drug is ‘reasonable and necessary’ for Medicare beneficiaries. An item or service is considered ‘reasonable and necessary’ if it is safe and effective, not experimental or investigational, and appropriate for use in Medicare beneficiaries.^67^ CMS then makes a National Coverage Determination (NCD) stating whether an item or service is covered by CMS. In the absence of an NCD, local contractors that pay Medicare claims make coverage decisions on a case-by-case basis. Under CMS’ CED program, drugs, devices, and services may be covered conditional on limiting access to clinical trials or with the collection of additional data to inform a reassessment. Predominantly, CMS has used CED agreements for medical devices. Self-administered drugs are not eligible for CED.^68^ CMS’ CED program does not use contractual agreements, and has been criticized for challenges with implementation, ethical concerns, duplication of post-market trials required by the US FDA, design flaws, and challenges with data collection and analysis.^69^

The recent authorization of aducanumab has brought renewed attention to CMS’s CED program for drugs authorized with limited evidence. On Jan. 11, 2022, CMS announced that aducanumab would be covered under the CED program, only when accessed through CMS-approved clinical trials.^70^ This decision has been highly controversial, largely due to the underwhelming results of one of the pivotal trials, which failed to meet its primary endpoint.^71^ Per the NCD, Medicare patients will be eligible to receive coverage of the drug, related services, and other routine costs, such as PET scans, if required by the clinical trial protocol.^72^

Coordinating regulatory and reimbursement decisions avoids duplication of work and evidence gaps. Like the US, Canada’s reimbursement decision-making processes are distinct from regulatory authorization decisions. When regulatory decisions are made on less mature clinical evidence, there is a downstream effect on reimbursement decision-making. Regulatory decision-makers need to be equipped to make reimbursement decisions with less mature evidence. Additionally, if both the regulator and the payor require post-market trials or data collection, there could be unnecessary duplication, therefore efforts should be made to coordinate. CMS’s decision to reimburse aducanumab within existing clinical trials rather that requiring a new clinical trial limits duplication.

Due to the variety of public and private payors in the US, it is possible that multiple overlapping, and perhaps contradictory MAAs are used for the same drug and indication. This represents an unnecessary duplication of agreement drafting and negotiation and may lead to conflicting or incongruent results. Additionally, by limiting RSAs to specific payors or institutions, access to treatment may differ between patients based on insurance coverage and location. Ideally, MAAs would be centrally negotiated to maximize negotiation and buying power, pool data resources for the most robust analysis, and ensure equitable patient access.

#### II.C.5. Israel

Israel has used MAAs as a mechanism for introducing new technologies into the public health system since 2014. Israel’s system for introducing MAAs is unique compared to previously reviewed jurisdictions in that a third party is used to mediate the interaction between payors and manufacturers. Each year, the Ministry of Health publishes a call for proposals for new health technologies to be added to the Health Services Basket, the list of health services covered by the public health system. To address value uncertainty and information asymmetry, the MAA Team mediates negotiations between the pharmaceutical company and the health plans, which occurs prior to deciding whether the health technology will be added to the Health Services Basket. This process is separate from the procurement negotiations. The MAA team first identifies health technologies that will have a higher chance of being listed with an MAA, such as where there is uncertain clinical benefit or a high level of public expenditure. The pharmaceutical company then drafts an MAA, usually a dose-capping scheme. The health plans are asked to provide information on the estimated number of patients that will use the technology to permit the MAA team to estimate the cost of listing the health technology. The MAA team then negotiates between the two parties to finalize the terms of the agreement. Once a technology is included in the Health Services Basket, its status is fixed, providing certainty for the manufacturer that the health technology will not be removed after the agreement concludes. Only the price may be altered following the expiry of the agreement.^73^

Israel can implement MAAs because of its robust health data system. All healthcare providers in Israel use electronic health records, and the entire Israeli population is covered by the state health care system, so collecting and reviewing administrative health data for the purposes of assessing agreements, and post-market surveillance more generally, is feasible and easily adopted within existing administration processes.^74^ As previously discussed, Canada’s health data infrastructure requires significant investment and modernization before it can be fully leveraged for MAAs.

Unique features of Israel’s approach are worthy of consideration. First, Israel involves a third party to mediate the negotiation process may help progress the negotiation and implementation of RSAs. Canada currently has third-party organizations that could serve a similar role between pharmaceutical manufacturers and provincial drug plan decision-makers, including CADTH and pCPA. Third-party involvement can also help to ensure that MAAs are used appropriately if one of their functions is to identify potential candidates for MAA consideration. Second, Israel decides whether an MAA is appropriate to pursue prior to making the listing decision, which may improve negotiations by allowing decision-makers to consider accurate information about the conditions under which the drug would be reimbursed. Additionally, pharmaceutical companies are encouraged to draft and negotiate MAAs, because it may improve their chances of a positive reimbursement decision. Negotiating the terms of the MAA prior to the listing decision provides the payors with leverage to negotiate more favourable agreements. In Canada, pCPA does negotiate listing agreements prior to final reimbursement decisions by the provinces; however, CADTH makes reimbursement recommendations without any information about potential MAAs. If MAAs were negotiated prior to reimbursement reviews, more accurate cost-effectiveness and utilization could inform the recommendation.

Third, Israel’s approach is unique in that it focuses on the quantity of health technology use rather than outcomes. While this approach is less administratively complex, it does not provide a built-in opportunity for post-market evidence development. However, considering the current barriers and complexities associated with post-market data collection, MAAs that focus on utilization rates rather than outcomes may be an effective interim option to limit expenditure of expensive new health technologies with uncertain clinical- and cost-effectiveness, while alternative options for post-market data collection are developed and implemented.

Israel’s listing decisions are permanent, providing certainty to the manufacturers that their products will remain funded, though the price may be altered. This approach mitigates some of the political and practical concerns associated with delisting or disinvesting drugs after the expiry of an MAA and preserves patient access if post-market evidence does not confirm clinical benefit. This approach also promotes cost-savings by linking price to level of evidence. However, this approach may not impact drug utilization, resulting in inappropriate use of drugs with unconfirmed clinical benefit. This approach is worth considering in the Canadian context to avoid complications associated with delisting but should be accompanied by physician and patient education to promote appropriate use.

## III. PART II

### III.A. Legal Barriers to Governance of MAAs

Implementing MAAs requires comprehensive governance and administrative structures that conduct negotiations, oversee agreements, make decisions about which drugs should be reimbursed through MAAs rather than traditional reimbursement decision-making processes, and make the final listing decisions following analyses of data collected during the term of the agreement. Canada’s complex and fragmented health and legal structures and systems pose unique challenges for governing MAAs, including government jurisdiction over health care, judicial oversight of MAA decisions, application of administrative law principles, and funding. Barriers limit governance options, including scope (federal or provincial) and nature (public or private). Finally, governance structures have implications for dispute resolution, accountability, and implementation.

#### III.A.1. Constitutional Law

In Canada, the *Constitution Act* (1867) divides the power to pass laws and regulate on different subject matter, known as the heads of power, between federal Parliament and provincial/territorial legislatures.^75^ While some heads of power clearly fall to one level of government, others, including health, are more complicated. The Supreme Court of Canada has ruled that health is an area of concurrent jurisdiction, meaning both levels of government can pass health legislation, depending on the focus of the legislation.^76^ The heads of power that grant law making authority to Parliament include its power over criminal law, which enables it to regulate or prohibit drugs, diagnostics and other health-related products and food. Parliament also has exclusive jurisdiction over taxation and spending powers, patents, aboriginal health services, quarantine (public health), and the peace, order and good government clause. While the imposition of the philosophical and societal contours of the health system, therefore, is in the hands of Parliament, the administration of healthcare is largely within the powers of provincial and territorial legislatures, stemming from three provisions in the *Constitution* that cover: the establishment, maintenance and management of hospitals (s. 92(7)); property and civil rights (s. 92(13)), which has been interpreted to include the regulation of professional services, health professionals and health^77^; and matters of strictly local or private nature within the province (s. 91(16)).^78^ As a result, jurisdiction over health in Canada is overlapping and oftentimes confusing, but public health insurance programs largely fall within the jurisdiction of provincial and territorial governments.

Any federal effort to control or influence provincial drug coverage programs, such as a federal initiative to implement MAAs, may be vulnerable to a constitutional challenge on the basis that it intrudes on provincial jurisdiction. The federal government could enact a federal Pharmacare program that would minimize the potential for jurisdictional challenges by relying on one of the following legal mechanisms. First, the doctrine of incorporation by reference permits one level of government to pass legislation incorporating legislative text enacted by the other level of government. Using this approach, the federal government could pass legislation setting technical standards for implementing an MAA governance structure or funding terms, which provinces could then be incorporated into provincial legislation by reference. This approach allows greater flexibility, as the incorporated document can be amended as needed, and any amendments are automatically incorporated into any legislation that references it.^79^ Similarly, a provincial government could enact a statute entrusting its implementation to a federal agency or body, while retaining jurisdiction. Through this type of constitutional maneuver, a provincial government could authorize a federal government agency to administer MAAs on its behalf. For example, the provinces have entrusted authority over blood supply to Canadian Blood Services through a memorandum of understanding. However, suboptimal adoption of such delegated power may exacerbate interprovincial inequities, for example, if one or more provinces refused to participate, resulting in ‘policy doughnuts’. This mechanism also leaves questions of funding unanswered.^80^ Considering vocal opposition expressed by some provinces to the adoption of a national Pharmacare program because of the intrusion on provincial jurisdiction,^81^ the adoption of a federally managed access governance structure would likely be suboptimal.

#### III.A.2. Administrative Law

Administrative law obliges governments to account for their actions and ensures that institutional decision-making is conducted in a fair and impartial manner, within the statutory mandate delegated to the decision-maker.^82^ There are two primary administrative law concerns that may arise under MAAs. First, a drug manufacturer could seek review of a decision made under an MAA, including a refusal to reimburse a drug in a MAA, a subsequent decision to extend, cancel, or suspend a MAA, or the findings of a reanalysis. Second, a patient could challenge a decision to exclude them from accessing treatment under an MAA or withdrawing them from an MAA.

Whether these avenues for review of decisions made under an MAA are available depend on the governance structure adopted. Administrative law typically applies only to government bodies and institutions, but an Ontario Court confirmed that as a non-profit federal corporation, CADTH was considered part of the machinery of both the federal and provincial governments, and its conduct was subject to judicial review.^83^ This suggests that any quasi-federal organization created to coordinate implementation of MAAs across provinces would likely be subject to judicial review. Similarly, if MAAs are implemented by provincial public drug plans, it is likely that, regardless of governance structure, decision-makers will be found to be public bodies and therefore subject to administrative oversight and judicial review.

When judicial review is available, an individual or organization may challenge a decision made by an administrative decision maker based on the content of the decision (substantive review) or the process by which the decision was made (procedural fairness). Substantive review allows courts to consider the content of a decision to determine whether it was sufficiently erroneous to send it back for reconsideration, using the appropriate standard of review, which is generally whether the decision was reasonable. In a minority of cases, Courts may apply the higher correctness standard if there is express legislative intent or if the rule of law requires it. The latter instances may arise with respect to constitutional questions, general questions of law of central importance to the whole legal system, and questions regarding jurisdictional boundaries between administrative bodies.^84^ It is likely that a reasonableness standard will be applied to any decisions made by an MAA governance body, including the decision to implement, terminate, or extend an MAA. If an MAA organization is created by legislation, the content of that legislation could stipulate that the correctness standard applies, although that outcome is unlikely.

An application for judicial review may also be brought on the basis that the administrative decision-making process was not fair. Procedural fairness is a vague concept in administrative law; no universal procedures exist to ensure fairness. Instead, flexibility is afforded based on the context of the decision. Often, to provide clarity, the administrative decision maker’s enabling statute will set out rules of procedure, which make it easier to determine unfairness if the rules are not followed. If the rules of procedure are not codified, the decision maker may determine the procedure on a case-by-case basis, but the procedure should nonetheless be consistent and predictable. The Supreme Court of Canada listed five non-exhaustive factors relevant to determining the content of duty of fairness:

1. The nature of the decision being made and process followed in making it.
2. The nature of the statutory scheme and the terms of the statute pursuant to which the body operates.
3. The importance of the decision to the individual or individuals affected.
4. The legitimate expectations of the person challenging the decisions and
5. The choices of procedure made by the agency itself.^85^

Judicial review based on unfairness in decision-making processes was one of two legal bases for challenge identified by in-house legal advisors when the Canadian Coordinating Office for Health Technology Assessment (CADTH’s predecessor) was chosen to house the permanent CDR.^86^ As a result, great care was taken in developing the procedures to ensure that all stakeholders were treated fairly.^87^ Similar care should be taken in developing MAA decision-making processes and governance structures to avoid claims of procedural unfairness.

Previous case law on reimbursement decisions could provide some insight into the administrative law issues relevant to MAAs. However, because administrative tribunals only have jurisdiction to hear matters within the authority granted to them by statute, administrative law cases to date have largely involved applications for judicial review of decisions derived from processes enshrined in legislation. For example, some provinces have legislation providing processes for reimbursing out-of-country health services and determining coverage of insured persons.^88^ The most common form of judicial review related to these provisions is for refusal of reimbursement or coverage. These cases are not particularly instructive, except for the lesson that judicial scrutiny will apply to procedural fairness, including transparency of reasoning for the decision.^89^

In addition to the determination by an Ontario Court that CADTH is subject to procedural fairness review,^90^ the Quebec Court of Appeal has held that decisions to delist a drug may require the Minister to respect certain minimum standards of procedural fairness.^91^ However, current HTA processes may fall short of procedural fairness requirements.^92^ As such, developing and adhering to clear procedural guidelines for MAA decisions, including initiating negotiations, reanalysis processes, and withdrawal procedures is necessary to minimize legal challenges from drug manufacturers. Evidence to date is less clear on whether patients may also have standing to raise concerns of procedural fairness. Generally, a duty of fairness is owed to individuals whose rights or interests are affected by the decision.^93^ For example, patients might be successful in claiming that HTA processes breach the duty of fairness owed to patients by not providing sufficient opportunity for patient input into decision-making,^94^ and a duty of procedural fairness may be owed when a reimbursement or coverage decision is specific to an individual.^95^ As a result, special attention should be paid to developing procedures for individual eligibility and continuation assessments.

In contrast, it is widely accepted that *policy* decisions to fund or not fund a treatment for a patient population are not subject to procedural fairness requirements.^96^ It is unclear whether decisions made to negotiate and implement MAAs (ie. deciding to initiate MAA negotiations, eligibility assessments, and reassessments) would be subject to substantive review, or whether they would be deemed policy decisions. Furthermore, while drug manufacturers generally have standing to seek judicial review of reimbursement decisions, it remains unclear whether individual patients or patient groups would have standing to seek judicial review of substantive decisions related to MAAs. Even if standing was granted, in highly technical contexts, such as HTA, courts are likely to afford significant deference to reimbursement decision-makers.^97^

In any case, the possible success and impact of judicial review applications will depend on the context, including the applicable statues and regulations, the decision process, and the facts of the case. It is likely that to implement MAAs, new legislative provisions and/or regulations will need to be enacted. The authority granted to relevant decision-making bodies set out in the legislative scheme will determine the scope of judicial review available.

### III.B. Legal Design and Implementation Considerations

In this section, we discuss the legal and ethical enablers and barriers for the design and implementation of MAAs, including equity and access; research ethics and consent; legal and regulatory uncertainty, patient challenges based on the *Canadian Charter of Rights and Freedoms* (the *Charter*), contracting, health data collection, privacy and confidentiality laws, and provincial reimbursement decision-making processes. These topics may limit or guide the design and implementation of MAAs or MAA programs.

#### III.B.1. Equity and Access

The introduction of MAAs may introduce novel equity and access concerns or exacerbate existing ones. Delisting or revising terms of access of funded drugs under an MAA may raise ethical and legal concerns. Patients may have come to rely on an MAA-directed treatment or may have forgone alternative treatment options to participate in the MAA. Changing access may, in some cases, be risky for patients or result in unfairness. To mitigate this concern, MAAs could include grandfather clauses or other access routes secured, such as compassionate access programs. Legal uncertainty exists about whether physicians can withdraw treatment without the patient’s consent; however, continued access may not mean that the drug would have to be reimbursed by a public drug plan.^98^

MAAs may also exacerbate concerns about equitable access to drugs in Canada. Priority populations, including Indigenous, racialized, and rural populations already face barriers in accessing drugs. Programs need to be designed so that priority populations do not bear a disproportionate risk and are not excluded from benefits under MAAs. Inequities in access also derive from Canada’s patchwork of public and private drug plans. Because health care decisions are made by each province/territory, MAAs may make some drugs available in some jurisdictions and not others, contributing to the ‘free-rider’ problem or ‘whipsawing’,^99^ and limiting access under MAAs to major urban centres may deepen the urban–rural health divide.

Existing equity-promoting initiatives may influence the implementation of MAAs. For example, Jordan’s Principle ensures all First Nations children living in Canada can access the products, services, and supports needed. Jordan’s Principle arose out of a 2016 Canadian Human Rights Tribunal decision that found the Government of Canada’s approach to services for First Nations children was discriminatory.^100^ Jordan’s principle ensures that Canada must make a decision on individual requests for supports and services for First Nations children within 12–48 hours of receiving a completed request, or within 12 hours for an urgent case.^101^ Under Jordan’s Principle, eligible First Nations children may be able to receive treatment with a drug or access to a diagnostic test that is otherwise limited in scope under a MAA. For example, in Jan. 2021, the federal government agreed to pay the full cost of zolgensma for a First Nations toddler to treat spinal muscular atrophy type 2. At the time, zolgensma was not reimbursed by any public drug plan in Canada.^102^

#### III.B.2. Research Ethics and Consent

MAAs operate at the intersection of clinical care and research, which are governed by distinct legal and ethical frameworks. While clinical research is governed largely by the Tri-Council Policy Statement: Ethical Conduct for Research Involving Humans (TCPS2)^103^, clinical care is governed by liability law and professional regulatory bodies. Whether data collection efforts under an MAA are considered research will therefore have implications for ethics oversight. Data collection may rely on administrative health data, patient registries, and other secondary data sources, or it may rely on prospectively designed clinical studies. The latter would be considered research, because patients would be required to undergo procedures, tests, or assessments that are not clinically necessary, but required to meet evidence generation requirements under an MAA. Whether the former would be considered research would be context dependent, however TCPS2 addresses health data and secondary use.^104^

TCPS2 covers research in institutions and organizations that are eligible to receive federal research funds.^105^ Such research requires approval and oversight by an institutional research ethics board (REB). TCPS2 requires robust consent mechanisms to enable patients to understand the potential risks and benefits of the treatment. Analyses that rely on secondary data sources, such as health administrative data, are usually categorized as research and therefore still subject to REB oversight. Exempted activities from the scope of TCPS2 include those characterized as quality assurance, program evaluation, and quality improvement.^106^ However, the activities directed by MAAs will straddle research and quality improvement, meaning that most will fall within the scope of the TCPS2.

Clinical care provided within an MAA will be subject to the common-law torts of negligence and battery, as well as breach of fiduciary duty, human rights claims of discrimination, and oversight by provincial professional regulatory bodies, including claims of professional misconduct. Though there is some overlap with TCPS2 principles, differences exist in consent and data requirements. As a result, it may be challenging to determine which ethical principles, laws, and policies apply to activities specified by MAAs.

Whether data collection under MAAs is classified as research has implications for consent. Consent requirements are more stringent for research, requiring disclosure of all risks, while physicians have some latitude to use judgment in disclosing risks when seeking patient consent for clinical care.^107^ However, a further concern arises if access to treatments under an MAA is limited only to patients who are willing to share their data or participate in medically unnecessary, potentially risky procedures; such a requirement could be considered coercive.^108^ Coercion could be mitigated by providing access to patients even if they do not consent to additional procedures or data collection.

#### III.B.3. Legal and Regulatory Uncertainty

Many of the laws, regulations, and policies relevant to the implementation of MAAs are currently being revised and new frameworks for drugs and medical devices are being discussed and developed. Here, we discuss some reforms that may impact the design and implication of MAAs.

First, the 2019 federal budget announced funding to establish a Canadian Drug Agency Transition Office and the development of the Drugs for Rare Diseases Framework (DRDF). Currently, the scope and impact of the DRDF is unknown, but it could have significant implications for the regulation and reimbursement of drugs in Canada.^109^ The 2019 federal budget also included funding to create a national formulary managed by an arms-length organization, the Canadian Drugs Agency.^110^ A national Pharmacare program would enable the adoption of MAAs; however, significant uncertainty remains as to the scope and administration of national Pharmacare. In March 2022, the Liberal Part of Canada and the New Democratic Party reached a supply and confidence agreement, in which the parties agreed to prioritize progress towards national Pharmacare by passing federal legislation by the end of 2023.^111^

Second, the pan-Canadian Advisory Panel on a Framework for a Prescription Drug List has undertaken to create a framework for developing a pan-Canadian prescription drug list, including recommending an initial list of included drugs, mechanisms for adding, and removing drugs from the list. Recommendations were expected to be finalized by Spring 2022. However, the recommendations are non-binding. Additionally, the scope of the Advisory’s Panel is limited, and specifically excludes consideration of potential governance structure for implementation of the formulary and financing the formulary.^112^

Another pan-Canadian strategy in the works is the pan-Canadian Health Data Strategy Expert Advisory Group. Established in 2020, the Advisory Group is tasked with modernizing health data collection, sharing and interoperability, streamlining and updating the approach to privacy and access for the digital age, and clarifying accountability, sovereignty, and health data governance to change the way governments share health data.^113^ The potential impact on health data sharing for the purposes of MAAs more broadly is unknown.

Third, CADTH is taking over the Drug Safety and Effectiveness Network, relaunching the program as the Post-Market Drug Evaluation (PMDE) Program in Sep. 2022. The purpose of the PMDE Program is to respond to queries from federal, provincial, and territorial governments regarding post-market drug safety and effectiveness. The goals of the PMDE Program include enhancing post-market query response capacity and capability and coordinating access to post-market drug information.^114^ The increased capacity to address post-market uncertainties offered through the PMDE program could be leveraged for MAAs and conditional drug authorizations.

Finally, several relevant regulatory reforms are under development at the federal level. New regulations are being developed that empower the Minister of Health to place terms and conditions on drug and device authorizations. Terms and conditions enable the collection of post-market information and may be used to adjust labels and licenses. Amendments are also under development to rely on foreign regulatory decisions, improve transparency, create new enforcement powers, and modernize environmental risk assessment requirements.^115^ Since these regulatory amendments are still under development, their implications for drug regulation and post-market evidence collection are unknown. Additionally, the *Food and Drugs Act* was amended in 2019 to provide for a regulatory framework for ATPs; however, regulations have yet to be implemented.^116^ Again, the specifics are unclear and the implications unknown.

Changes to the federal privacy law framework could also have implications for MAAs. In Nov. 2020, Bill C-11, the *Digital Charter Implementation Act, 2020* was introduced into Parliament.^117^ If enacted, it would significantly reform federal sector privacy legislation and may result in provincial privacy legislation amendments to align with the federal legislation. Such amendments could have implications for data collection, use, storage, and sharing practices within and across health care institutions. However, when the 43rd Parliamentary session was dissolved, progress on Bill C-11 halted. Bill C-11 has not been reintroduced to Parliament in the new session, so the future for this initiative is uncertain.

In addition to proposed and upcoming changes, the scope and interpretation of some laws and regulations are currently unclear, creating regulatory uncertainty for the design and implementation of MAAs. For example, *Vanessa’s Law* amended the *Food and Drugs Act*, empowering the Minister of Health to recall drugs, order a label change, require information, disclose confidential business information, require assessments of therapeutic products, and require tests, studies, or monitoring.^118^ To date, there has been little judicial consideration of *Vanessa’s Law*, but aspects of the legislation that could be subject to judicial interpretation, including the prerequisite of whether the product in question presents ‘a serious risk’. Serious risk is not defined; instead, determinations of serious risk are intended to be made on a case-by-case basis. Enforcement of these new provisions is also uncertain. The guidance documents state that the new powers are intended to be used as a last resort, only if parties are not willing to comply voluntarily.^119^

#### III.B.4. Application of the Charter and Human Rights and Genetic Non-Discrimination Legislation

An individual that is excluded from access to treatment under an MAAs may challenge their exclusion on the basis that it is discriminatory and/or violates rights protected by the *Charter*. The *Charter* allows individuals to challenge any government law, policy, action, or inaction believed to violate the rights and freedoms it protects. As a result, if the federal or provincial governments pass legislation to implement MAAs, it must comply with the *Charter*. Additionally, the *Charter* applies to administrative decisions made pursuant to an enabling statute, such as determining which patients can access reimbursed treatments pursuant to a MAA. The *Charter* has been found not to apply to hospitals in their routine operations; however, hospitals must comply with the *Charter* when delivering medical services to the public pursuant to law and government policy.^120^

Health care coverage decisions can be subject to *Charter* litigation, typically on the basis of infringing the right to equality or the right to life, liberty, and security of the person. Both are difficult to litigate successfully, and previous jurisprudence has largely focused on access to services, such as autism services.^121^ Under section 7 of the *Charter*, patients may argue that a law or government action that removes or narrows medical options contravenes their right to life, liberty and security of the person. This avenue is only available where legislation or administrative decisions expressly prohibit patients from obtaining certain treatments or medical services, such as was the case in *Chaoulli* and *Cambie Surgeries Corporation*, which both related to the prohibition of accessing private medical services.^122^ This legal avenue may be contemplated if specific legislation is enacted restricting access to health technologies under MAAs in some manner or an individual is excluded from accessing or continuing to access a treatment provided under a MAA. However, since reimbursement decisions do not preclude a patient from paying for a drug via other means, such as private insurance or out of pocket, section 7 is unlikely to be engaged. Courts have generally shied away from recognizing positive obligations under section 7 of the *Charter*^123^, making it an unlikely pathway for patients to argue that the government has an obligation to provide access to a specific treatment.

Section 15 of the *Charter* provides a right to equality and may provide a means for patients to challenge a government’s decision not to fund a specific treatment or medical service on the basis that it is discriminatory. Similarly, federal and provincial human rights legislation protects individuals from discrimination. While the *Charter* applies only to government actors, provincial human rights legislation applies to both private and public actors. Additionally, *Charter* rights must be enforced in court, while human rights complaints can be heard by a tribunal or filed with the appropriate human rights agency or commission. In both scenarios, the court or tribunal must be satisfied that the law creates a distinction between patients who do have access and those who do not based on an enumerated or analogous ground^124^ and the distinction must create a disadvantage by perpetuating prejudice or stereotyping.^125^ If a government enters into an MAA that reimburses a health technology for some populations, but not others, decision-makers may be exposed to *Charter* litigation or human rights complaints. However, while a patient is unlikely to successfully challenge an MAA for failure to fund a specific treatment,^126^ its eligibility criteria should be carefully designed to avoid discrimination. A finding of discrimination is unlikely if eligibility criteria are based on evidence of safety and efficacy. For example, the British Columbia Court of Appeal found that it was not discriminatory to fund mammograms and Pap tests for women, but not prostate-specific antigen testing for men, because the latter was not supported by evidence.^127^

The *Genetic Non-Discrimination Act* (*GNDA*) may also impact the negotiation of MAAs that cover drugs reliant on a genetic test. The *GNDA* prohibits requiring an individual to undergo a genetic test or disclose the results of a genetic test to access goods or services or enter a contract. The *GNDA* does not apply to physicians, pharmacists or health care practitioners, or any person who is conducting medical, pharmaceutical, or scientific research.^128^ Whether this legislation would apply in the context of an MAA is unclear. A strict reading of the legislation suggests that a public drug plan requiring a person to undergo a genetic test and to share the results of the test to access medical services would be a prohibited act. However, a modern purposive approach to statutory interpretation requires a statute to be interpreted consistent with its intended purpose. While a full analysis of this issue is beyond the scope of this paper, the result would likely hinge on the distinction between access to health services and reimbursement of health services.

As written, the *GNDA* permits health practitioners to require genetic tests to determine appropriate clinical treatment; however, it does not reference reimbursement. It can be inferred that Parliament’s intent in carving out this exception was to maintain clinical freedom. A physician’s ability to practice medicine is independent from government funding of medical services and products. Statutory interpretation principles would then imply that if Parliament intended the *GNDA* to exclude provincial governments and their agencies from the provisions of the *GNDA*, it would have done so. This issue has been considered more broadly outside the context of the *GNDA*, and the authors of one review article suggest that requiring genetic tests to access treatment should not be problematic if the tests are validated, contained in the label, and the degree to which results are predictive of efficacy is sufficient.^129^ However, Health Canada regulation of companion diagnostics is in its infancy and the labeling may reference the presence of a specific mutation, eg *HER2+*, *PD1*, rather than a prescribe a specific test. Many such genetic tests are laboratory developed, whose regulation falls under provincial regulation of the laboratory, rather than Health Canada regulation of the test kit.

#### III.B.5. Contracting

In all provinces but Quebec, contracts are largely governed by common-law, also known as judge-made law, and some legislation, such as *Sale of Goods Acts*, because the provinces have Constitutional jurisdiction over matters of a private or local nature.^130^ As a result, interprovincial differences in contracting practices and interpretation may complicate the ability to standardize MAAs across multiple jurisdictions. For example, privacy and confidentiality clauses will need to reference applicable provincial legislation, which may impose different requirements.

Types of clauses that deserve special attention when drafting MAAs include: eligibility criteria; stopping criteria; termination, extension, and renegotiation clauses; and dispute resolution clauses. A lesson on drafting eligibility criteria, which was the subject of an MAA dispute in the UK, is to consult appropriate stakeholders to ensure that eligibility is compatible with existing clinical practices.^131^ Similarly, eligibility criteria should be based on clinical evidence and outstanding uncertainties to avoid potential claims of discrimination. Clauses on procedures and processes for terminating, extending, or renegotiating the agreement also require special attention. Such terms should be clearly linked to outcomes, to avoid ‘dangling’ MAAs that are continuously extended because evidence development is poorly designed, sluggish, or inconclusive. Clear dispute resolution procedures should be included to adjudicate the inevitable disputes about data results, analysis methods, and outcome metrics.

While experience with PLAs in Canada provides useful experience to introduce MAAs, MAAs are more complex than simple listing agreements, and may require the implementation of several agreements in addition to PLAs, including patient consent documents, data sharing agreements, and funding agreements.

#### III.B.6. Health Data Collection and Analysis

Health data collection is integral to the operation of MAAs. Existing mandatory and/or discretionary mechanisms for data collection, including post-market surveillance mechanisms, administrative health data held by government ministries and health systems, patient registries, and clinical trials are unlikely to be sufficient to support MAAs. Data gaps may hinder data collection for analyses to resolve clinical uncertainties. For example, electronic health records uptake has been uneven, so patient charts are often held in clinician offices, although uptake of electronic health records continues. Often required data elements for analysis are in physician notes, making data abstraction time-consuming and inaccurate. Additionally, data and privacy laws, regulations and policies may pose challenges for data collection and analysis required under an MAA.

Mandatory post-market surveillance mechanisms exist for all drugs authorized by Health Canada, which enable drug manufacturers to report data on adverse drug reactions. Serious adverse drug reactions must be reported immediately (within 15 days).^132^ Drug manufacturers must prepare and submit annual summary reports containing all information on adverse drug reactions and determine whether there has been a significant change in the benefit–risk profile of the drug.^133^ Health Canada can also request issue-related summary reports at any time to assess the safety and effectiveness of the drug.^134^ These initiatives are largely focused on safety signals, so their relevance for addressing clinical- and cost-effectiveness uncertainties is likely minimal.

*Vanessa’s Law* provides Health Canada with mechanisms to collect post-market evidence. It empowers the Minister of Health to require drug manufacturers, researchers, and individuals to submit information believed to be necessary to determine whether the health product presents a serious risk of injury to human health. Similarly, it permits the Minister of Health to require drug manufacturers to conduct assessments of authorized products upon reasonable grounds that the benefits or risks associated with the therapeutic product are significantly different than when the authorization was issued. The Minister of Health may also order drug manufacturers to compile information, conducts tests or studies, or monitor experience based on reasonable grounds that there are significant uncertainties relating to the benefits or harms of the drug.^135^ These powers are intended to be used only as a last resort if the parties are not willing to comply voluntarily.^136^ Additionally, while *Vanessa’s Law* provides the authority for Health Canada to collect enhanced post-market drug data, these powers require a high threshold to be met, which is unlikely to be triggered by cost-effectiveness uncertainties.

Administrative health data are another potential source to support analyses required by MAAs. However, their use for MAAs may be limited, depending on the complexity of the research questions to be resolved and jurisdictional constraints. Health administrative data gaps include mismatched data points or formats, anonymized data that prevent long-term follow up or linking multiple databases, and restrictive data sharing practices and regulations.^137^ Additionally, some provinces or institutions may have greater capacity to use administrative data for the purpose of an MAA. For example, a demonstration project determined that outcomes-based agreements relying on overall survival or time to next treatment as outcomes could be operationalized within Alberta’s existing administrative health data systems.^138^ As a result, depending on provincial capacity and the governance structure, designing MAAs to utilize existing administrative health structures may be feasible.

Separate from its regulatory functions, Health Canada partnered with the Canadian Institutes of Health Research (CIHR) to establish the Drug Safety and Effectiveness Network (DSEN) to increase evidence on drug safety and effectiveness and increase capacity to undertake post-market research. In 2021, CIHR announced that CADTH would be taking over and relaunching DSEN as the Post-Market Drug Evaluation (PMDE) Program in Sep. 2022. The PMDE Program will launch and coordinate a network to leverage Canadian expertise and deliver post-market safety and effectiveness evidence for use in Canada.^139^ Once operational, governments, HTA bodies, pCPA, and PMPRB will be able to submit queries to the PMDE Operations Centre regarding post-market drug safety and effectiveness. The Operations Centre will serve as a hub to connect people and will not access or hold data. After conducting a feasibility assessment, the Operations Centre will identify an appropriate network response team for the query. After conducting evidence generation and analysis, the response team will then submit a report of findings to the PMDE Operations Centre and the customer.^140^ Though the PMDE Program does not specifically contemplate evidence generation for MAAs, it may provide an important supplementary source of health data.

Patient registries provide another data collection mechanism for the purposes of MAAs. Patient registries offer certain benefits, including the ability to tailor data collection for the disease and specific purpose of the MAA, the collection of patient reported outcomes and other measures not typically captured through routine care, and the ability to leverage relationships with patient stakeholders and existing registries, where available. Limitations of patient registries include the lack of control groups and the costs and resources associated with setting up patient registries for each MAA, including obtaining participant consent.^141^

Finally, data can be prospectively collected through formal clinical trials to answer outstanding or emergent clinical- and cost-effectiveness uncertainties. This approach is burdensome, in terms of cost, time, and ethical oversight, but has the potential to provide greater methodological rigor by including control arms and randomization. Integrating research into clinical practice can ameliorate some of the burden associated with formal clinical trials. Such an approach allows for data collected through routine care to be used to answer research questions, imposing less burden on patients and researchers. Currently, pragmatic clinical trials are generally limited to individual institutions or organizations because of barriers to data sharing, discussed in greater depth below. Additionally, there are outstanding ethical concerns and implementation challenges that must be addressed. For example, randomizing treatment protocols in a clinical care setting raises potential concerns of interfering with clinical judgment.

#### III.B.7. Reimbursement Decision-Making

The ability to list and delist health technologies are important features of MAAs; however, existing reimbursement frameworks may pose barriers to the implementation of MAAs. In accordance with the *Canada Health Act*, in-hospital drugs are financed by provincial and federal governments.^142^ Generally, in-hospital drugs are funded by hospital budgets and not by public drug plans. Coverage of take-home oral cancer medications varies across Canada as well.

Though most provinces/territories conduct their own analyses to decide which drugs to reimburse following CADTH (or in the case of Quebec, INESSS) recommendations, there can be considerable variation in the scope and detail of jurisdictional analyses. Each province public drug plans covering certain populations, including seniors, low-income individuals and families, and certain disease groups, such as diabetes and cancer. Ontario is the only jurisdiction that has a policy on reimbursing drugs within clinical trials.^143^ Interprovincial differences may hinder the adoption of pan-Canadian MAAs.

Additionally, although each jurisdiction has the authority to fund drugs unconditionally or only upon prior approval, each province uses different mechanisms to demonstrate whether clinical criteria have been met. Some provinces designate authorized prescribers, thereby reducing the burden of individual requests, but some require individual authorization, which could increase administrative burden for MAAs. For example, in British Columbia, PharmaCare has the capacity to verify whether clinical criteria have been met by reviewing prescription dispensation data. British Columbia also utilizes prescription exemptions and Collaborative Prescribing Agreements to exempt prescribers from submitting special authority request forms.^144^ In contrast, Ontario verifies that clinical criteria have been met for Limited Use Products by requiring prescribers to input a Reason for Use code confirming that treatment is within the specified circumstance.^145^ These different approaches to verifying clinical criteria prior to reimbursement will influence the design and adherence to MAAs.

Provinces other than Quebec have clear legislative provisions that permit the removal of drugs from public drug formularies for any reason, without notice. Such provisions would enable drugs to be delisted following a reassessment under an MAA.^146^ In Quebec, coverage for a drug may be terminated in certain circumstances, including when: INESSS recommends it; where the manufacturer does not comply with a condition or commitment provided for by regulation, a registration agreement, or a contract; when the sale price is greater than the maximum amount payable by the general plan; when a medication is the subject of a registration agreement; or, when the Minister is of the opinion that doing so is in the public interest. Notice must be published on the Ministry’s website. Delisting orders may maintain insurance coverage for patients who have already initiated treatment with the drug.^147^ There may therefore be an added burden using MAAs in Quebec. However, delisting drugs is often difficult in practice even if allowed by law and policy because of political pressure, logistics and the ethical and social challenges of withdrawing access from patients.^148^

### III.C. Legal and Policy Implications of MAAs

Other legal domains may be impacted by the implementation of MAAs, including those related to securities regulation; liability of governments, institutions, physicians, and drug manufacturers; competition law; employment law; and intellectual property. These legal domains are unlikely to impact the design and implementation of MAAs directly but may indirectly affect the legal rights and obligations of stakeholders, including physicians, hospitals, drug manufacturers, and patients. We discuss each in turn.

#### III.C.1. Securities Regulation

In Canada, securities are regulated at the provincial level. Securities regulation is concerned with the sale and transfer or securities.^149^ Though there are some exemptions, securities cannot be distributed without filing a prospectus, a comprehensive disclosure document providing information on the business and securities being offered.^150^ In addition, public companies are subject to continuous disclosure obligations. A public company must issue and file press releases when a material change in its affairs occurs, or when material information relating to its affairs becomes known to management.^151^ A ‘material change’ is a change that would reasonably be expected to have a significant effect on the market price of value of its securities.^152^ Breach of disclosure requirements can result in civil and administrative proceedings. Public companies may be liable to investors for damages resulting from misrepresentations in a publicly disclosed communication or failure to make timely disclosure.^153^

The implications of MAAs on securities regulation in Canada are uncertain. However, a federal inquiry in the US into post-market confirmatory trials required for drugs authorized under the Accelerated Approval process identified the potential for securities regulation concerns over appropriate disclosure of the status and existence of post-market clinical trial requirements.^154^ There has been one Supreme Court of Canada decision relating to securities regulation and pharmaceutical related disclosure. In *Theratechnologies Inc v 121851 Canada Inc*, 121851 Canada Inc. sought to launch a class action proceeding for damages claiming that information about the side effects of tesamorelin, the drug in question, and the US FDA’s questions about the side effects amounted to a material change, triggering disclosure obligations. The Court held that the content of a US FDA briefing to its advisory committee did not rise to the level of a material change, because Theratechnologies Inc. had already disclosed to its shareholders that it was monitoring tesamorelin’s side effects and provided results of clinical trials.^155^ As a result, companies may have an obligation to disclose the existence and nature of MAAs, as well as any relevant findings, including interim results, to their shareholders.

#### III.C.2. Liability

Liability commonly results from negligence, but special duties may arise depending on the parties involved, including government (federal and provincial), institutions (hospitals), clinicians, and drug manufacturers. Claims may originate from a patient who is injured by the drug or excluded from accessing treatment under an MAA. Alternatively, claims may originate from a drug manufacturer based on the decision not to enter into an MAA.

Canadian courts have limited government liability in the health sector. Federal government liability under an MAA is unlikely because provincial governments would most likely be responsible for entering into MAAs. Courts have found that Health Canada does not to owe a duty to consumers when it authorizes defective medical devices.^156^ It also does not owe a duty to individual consumers of drugs or devices because such a duty would conflict with Health Canada’s broader duty to the public. Similarly, courts have been reluctant to impose liability on provincial ministries of health. Decisions on which drugs to fund, and the conditions of reimbursement, are considered policy decisions and therefore exempt from liability.^157^

Health care institutions^158^ may be liable for patient injuries in some circumstances. Hospitals owe a duty of care to patients, and if a hospital breaches the standard of care, it could be liable for any resulting injuries (direct liability). Additionally, hospitals may be held liable for the conduct of their employees (vicarious liability).^159^ Institutional liability is most likely to arise when an institution has implemented a policy, and failure to adhere to that policy has led to a patient injury.^160^ As a result, if an MAA is implemented in a health care institution, that institution should implement appropriate policies and ensure they are followed. Patients can also sue laboratories and diagnostic facilities. For example, a laboratory could be sued for improper calibration of equipment or inaccurate interpretation of a diagnostic test, which could arise if a fault diagnostic test excludes a patient from accessing treatment under an MAA.

Liability for patient injury most commonly arises out of physician acts or omissions. Providing care under an MAA may introduce novel considerations for physicians to protect patients and avoid liability. For example, a physician may be liable in battery if a patient is treated without obtaining valid consent, or in negligence for failing to disclose material risks.^161^ For consent to be valid, physicians may be required to inform patients about the terms of the MAA, access to a treatment funded under an MAA even if they do not meet the eligibility criteria, the possibility of reimbursement being withdrawn or discontinued under an MAA, and other associated risks.^162^ Physicians may also be vulnerable to liability for withdrawing treatment without consent; however, Canadian law remains uncertain over whether physicians can unilaterally withdraw treatment without a patient’s consent, particularly where the treatment is lifesaving.^163^

Generally, drug manufacturers may face liability for defective design, defective manufacturing, and failure to warn. Failure to warn is most likely, although MAAs will not necessarily pose any additional risk of such a claim. It is established in Canadian law that a manufacturer of a product has a duty to warn consumers (through physicians) of dangers inherent in the use of its product. This duty is ongoing, so manufacturers must warn of dangers discovered even after the product is on market.^164^ If, during the course of a MAA, new risks are identified, the manufacturer may have an obligation to warn patients, including existing patients.

#### III.C.3. Competition Law

Competition law influences the environment in which drug manufacturers compete and consumers access drugs and other regulated medical products and services. Both the federal and provincial governments regulate competition through legislation and regulation.^165^ The federal *Competition Act* addresses both criminal conduct and civil or ‘reviewable’ conduct that can have an anti-competitive effect in the Canadian marketplace. The focus of the *Competition Act* is to eliminate activities that reduce competition. The *Competition Act* applies to all businesses operating within and between the provinces, and therefore is applicable to the sale and procurement of therapeutic products, but the Crown is only vulnerable to the *Competition Act* where an agent of the Crown is a corporation involved in commercial activities.^166^ The governance structure implemented for MAAs will determine the application of the *Competition Act.*

If the *Competition Act* applies, there are criminal provisions that regulate conduct, including conspiracy, bid-rigging, and criminal deceptive marketing practices. In addition, there are eight types of civil offences, including civil competitor collaboration, abuse of dominant position, price maintenance, refusal to deal, exclusive dealing, tied selling, market restriction, and mergers. Because competition law is chiefly concerned with regulating conduct between competitors, MAAs are unlikely to introduce novel anti-competition concerns. However, Canada’s current competition law framework may constrain the ability to negotiate and implement MAAs or may introduce novel considerations for stakeholders to be aware of. For example, if a request for tenders process is used to solicit bids for MAAs, as is currently the case in Alberta, this may introduce the possibility of bid-rigging (when parties agree not to submit a bid). However, nothing specific to an MAA request for tenders process increases the risks of bid-rigging. The same is true for other offences, including conspiracy to fix prices, allocate markets, or control output levels.

Other competition considerations include abuse of dominant position and restrictive practices. Abuse of dominant position occurs where entry of genetic competitors is inhibited or deterred to preserve market power.^167^ Offering rebates to insurers in exchange for using more expensive brand name drugs over cheaper generics, or otherwise influencing prescribing, could be considered an abuse of dominance. Rebates are not explicitly identified as a form of potentially abusive conduct under the *Competition Act* but may be an implicit form of predatory conduct.^168^ Generally, the types of drugs that will be considered for an MAA will still be under patent protection, avoiding this concern.

Restrictive practices, including exclusive dealing and tied selling may also be a concern under MAAs. Exclusive dealing occurs when a supplier requires or induces a customer to deal only, or mostly, in certain products.^169^ Certain practices under a MAA, such as including as a term of the agreement a requirement to deal mostly, or solely with a specific drug, may raise concerns of exclusive dealing. For example, an MAA may include terms to privilege the use of certain drug products over others, which, if found to have the effect of reducing competition or discouraging a firm’s entry into the market, may be found to be exclusive dealing. However, considering the interest of both the drug manufacturer and the payor to provide the drug, MAAs are unlikely to reach the threshold of requiring or inducing a customer to exclusively deal.

Tied selling occurs when a supplier, as a condition of supplying a particular product, requires or induces a customer to buy a second product or prevents the customer from using a second product with the supplied product.^170^ There is some evidence that tied selling is already a common practice in the pharmaceutical industry, for example, when a supplier provides a rebate or a discount for one product only if it is used in conjunction with a second product. This often occurs when a brand name manufacturer provides a rebate or discount to a drug plan for one drug only when used in combination with a second brand name drug, even though cheaper alternatives exist. While MAAs are likely to focus on the use of one drug product only, it is possible that MAAs could raise additional concerns about tied selling.

#### III.C.4. Employment and Labour Law

One of the major barriers identified in literature on MAAs is the administrative burden placed on physicians, nurses, and other staff to collect, log, and analyze patient data and obtain patient consent, if necessary.^171^ Adding new responsibilities not directly related to patient care may require policy changes, such as adding a new billing code for physicians to bill appropriately for their time. Additionally, the introduction of MAAs may introduce labour and employment law considerations. Employers are required to abide by applicable legislation, as well as any collective agreements. Health care is provincially regulated, so each province has labour legislation, and there are numerous involved unions with their own collective agreements. Changing the duties of unionized employees, such as nurses, may have legal consequences or require following procedures set out in the collective agreement. Additionally, bringing in non-union employees to perform work ordinarily done by the bargaining unit may result in a cease-and-desist order.^172^ It is beyond the scope of this document to review all applicable collective agreements. Labour laws and collective agreements should be consulted prior to assigning work required for MAAs. Differences between provincial labour laws and individual collective agreements may require tailoring human resources solutions to each province or institution.

#### III.C.5. Intellectual Property

In Canada, pharmaceutical intellectual property is protected by patents and data exclusivity. Patents provide the inventor of a drug or medical device to enforce their rights to exclude against others. In addition, the *Food and Drug Regulations* provides for a period of data exclusivity for eligible innovative drugs containing novel medicinal ingredients, preventing subsequent generic manufacturers from filing a submission to Health Canada based on a comparison to the innovative drug.^173^

Intellectual property considerations may be an important consideration when drafting the agreement. For example, the Pfizer-Israel COVID-19 Vaccine agreement included language clarifying that all data collected under the agreement would be owned by the Israeli Ministry of Health but that Pfizer had the right to use the data for research and development, regulatory submissions, and publications.^174^ Such a clause will likely be necessary to ensure that drug manufacturers can use the data collected under an MAA for specified purposes.

Additionally, exercising intellectual property rights of diagnostic tests could pose challenges for implementing MAAs. Until recently, medical diagnostic inventions were often deemed unpatentable. A 2020 guideline update altered the approach to determining the patentability of an invention such that medical diagnostics may be eligible.^175^ This policy change could incentivize the development of commercial diagnostic kits in Canada and impact the development and use of laboratory developed tests; patent rights may prohibit hospitals and health institutions from conducting similar in-house tests, instead requiring the outsourcing of diagnostic tests, typically at significant cost.

Under the *Patent Act*, the Commissioner of Patents is empowered to authorize compulsory licensing, the public, non-commercial use of a patent by the federal or provincial government, effectively bypassing patent rights if efforts have been made to obtain authority to use the invention without success.^176^ One case from the Children’s Hospital of Eastern Ontario (CHEO) highlights the uncertainty of compulsory licensing for diagnostic tests. Transgenomics, a US company, held patents for gene mutations associated with long QT syndrome. CHEO challenged the patent’s validity but before the courts could decide the matter, the parties settled on publicly disclosed terms, including a royalty-free licence of the patents to CHEO and other Canadian healthcare institutions for not-for-profit LQTS testing and research. While the settlement was viewed as a success, it prevented the courts from weighing in on the legal issues of compulsory licensing of personalized medicine and diagnostic patents.^177^ Negotiating licensing or other commercial agreements to implement diagnostic tests for the purposes of a MAA may add complexity to the process.

## IV. PART III

### IV.A. Governance Options for MAAs in Canada

Various governance structures could be used to deploy MAAs in Canadian jurisdictions, but these will be limited by Canada’s legal, regulatory, political, and geographical realities. Here, we consider policy options for governance, considering Canada’s constitutional framework and existing health infrastructure. The governance structure selected will depend on the scale and scope of the intended use of MAAs. Generally, there are three possible governance approaches for adopting MAAs in Canada: a fully federal organization, a quasi-federal organization, or provincial organizations.

### IV.B. Fully Federal (Canada Drug Agency)

The first option is to implement MAAs on a pan-Canadian scale by creating a federal organization or authorizing Health Canada to regulate MAAs. Because this would be an intrusion on provincial jurisdiction, the federal government would either have to argue that the regulation of MAAs falls under their constitutional jurisdiction or the provinces would need to formally delegate the authority to implement MAAs to a federal agency or body. For example, the federal government could argue that implementing MAAs appropriately falls into their authority to introduce legislation for peace, order, and good government (POGG), a residual power granted to the federal government to enact legislation in three scenarios: areas that the Constitution does not allocate to either level of government; emergencies; or areas of national concern.^178^

The most likely strategy would be to argue that implementing MAAs is a matter of national concern. Though national concern branch is rarely relied upon as justification, its use has recently been affirmed by the Supreme Court of Canada. In *References re Greenhouse Gas Pollution Pricing Act*,^179^ Saskatchewan, Ontario, and Alberta challenged the constitutionality of the *Greenhouse Gas Pollution Pricing Act* (*GGPA*). In a 6–3 decision, the Supreme Court of Canada held that the *GGPPA* was constitutional, on the basis that Parliament has jurisdiction under the national concern branch of the POGG power. However, it is beyond the scope of this paper to assess its successful use in the context of MAAs, and it is important to note that constitutional cases take years to resolve.

The federal government may alternatively argue that implementing an MAA program is a valid exercise of their criminal law power. The federal government can justify action as within the scope of its criminal law power, which the courts have held confers a preventative function to protect the public from harm.^180^ Protecting the public from unsafe or adulterated drugs is the justification by which the Federal government uses its criminal law power to authorize health technologies under the *Food and Drugs Act.*^181^ If the federal government were to introduce an MAA scheme via new legislation or an amendment to the *Food and Drugs Act* and *Food and Drug Regulations*, they would need to demonstrate that MAAs are necessary to protect the public from harm. A more recent case, *Reference re Assisted Human Reproduction Act*^182^ muddies the waters on the extent of the federal government’s criminal law power. The split decision demonstrated a deep lack of clarity regarding the line between criminal law and health, with half of the court adopting an expansive definition of the criminal law power and the other half arguing that the legislation in question was more appropriately categorized as related to health. The tiebreaking vote split the difference, upholding some provisions as properly within the federal government’s criminal law power, but striking down others as ultra vires.

It is difficult to conclude whether an MAA scheme could be justifiably enacted under the federal government’s criminal law power without knowing the specifics of the MAA policy. For example, it could be argued that it is within the federal government’s criminal law power to add a new category of drug authorization, akin to the NOC/c policy, that limits access to an authorized drug to patients enrolled in a patient registry, clinical trial, or other evidence generation programs, subject to reassessment, particularly if limited to circumstances where there are legitimate concerns or uncertainties for patient safety. Indeed, under the NOC/c policy, Health Canada has in some circumstances limited distribution of a drug to controlled access programs, demonstrating Health Canada’s willingness to limit market access beyond licensing. However, broader adoption of such an approach to address cost-effectiveness or even clinical effectiveness is likely to face significant resistance from industry, provincial ministries of health, physicians, and patients, who will argue that it is an inappropriate intrusion on the practice of medicine, which is within provincial jurisdiction.

A second strategy is to use administrative interdelegation, as described above, which would allow a provincial government to retain formal jurisdiction while permitting the federal government to take responsibility for the implementation of a policy or program.^183^ Provinces that do not wish to participate can opt out and retain their authority; however, this would result in inconsistent access and ‘policy doughnuts’.^184^

In sum, despite the potential benefits of adopting MAAs on a national scale, including efficiency and consistency of access, Canada’s constitutional framework imposes significant barriers to adopting this approach. Relying on the national concern branch or criminal law power would likely be subject to lengthy legal challenges, while delegation would require collaboration amongst federal and provincial governments and could result in inequitable access in non-participating jurisdictions. Additionally, a federal approach would require significant investment in data infrastructure and would be likely to face difficulties in navigating federal, provincial, and institutional health data and privacy laws and policies.

An example of the political and policy gridlock in health-related programs, one only needs to point to longstanding attempts to create a national Pharmacare program. While remaining a national priority, little headway has been made. Preliminary funding was announced in the 2019 budget to create a national formulary managed by an arms-length organization, the Canadian Drugs Agency.^185^ At the time of writing, no legislation has been introduced to move national Pharmacare forward. Should national Pharmacare ever come into existence, MAAs may be a natural extension.

#### IV.B.1. Quasi Federal Agency/Organization

A middle ground option is to leverage Canada’s existing quasi-federal organizational expertise to support the adoption of MAAs. To date, both CADTH and pCPA have expressed interest in MAAs or other similar approaches. For example, CADTH often recommends reimbursement conditional on lowering price or addressing evidence uncertainties. Similarly, pCPA negotiates PLAs on behalf of participating jurisdictions, but the final decision to enter into an agreement ultimately rests with each province. Under this approach, full authority would still rest with provinces to decide whether and how to implement MAAs, but pCPA and CADTH would provide analytic and decisional support. Alternatively, a novel quasi-federal organization could be created to support the design, implementation, and administration in participating jurisdictions by bringing together relevant stakeholders from each province. However, the limitation of quasi-federal agencies to only provide non-binding recommendations is a barrier, evidenced by the lack of MAAs implemented to date. In one case of which we are aware, pCPA displayed interest in negotiating an MAA, but negotiations were unsuccessful.^186^

To pursue this option, additional funding, training, and collaboration would be required for pCPA, CADTH, and responsible provincial authorities. Survey respondents indicate that CADTH is unlikely to have the resources to contribute meaningfully to MAA processes.^187^ To streamline the process, CADTH and pCPA could develop procedures to recommend MAAs and standardized options for types and features of MAAs. However, pCPA and CADTH would likely be limited to recommending the basic principles of the agreement; each province would need to tailor the agreement to local laws, regulations, and practices. As a result, MAAs could differ substantially between jurisdictions when negotiations are completed.

Data collection and analyses to resolve uncertainties under MAAs raise questions, such as whether data would be analyzed within each province or shared and analyzed in the aggregate. Province-specific collection and analysis would face fewer legal hurdles but would encounter methodological challenges, including small sample sizes and different rates of data accrual, which would complicate re-analysis. Data aggregation across jurisdictions would allow larger datasets to be leveraged but would create administrative and legal challenges in sharing patient data. Most provinces allow health data to be shared with patient–participant consent, or data may be deidentified prior to sharing with an arm’s length agency for analysis. Alternatively, each province has different requirements for the disclosure of personal health information without consent, so this solution may not be an option in all jurisdictions. For example, in Ontario, a health custodian is permitted to disclose health information ‘[t]o determine or verify the eligibility of the individual to receive health care or related…services…that are provided and funded by the provincial or federal government.’^188^ This type of provision may permit disclosure of personal health information to assess the eligibility of potential participants, however, it is not clear that this type of disclosure exemption would apply to disclosure for the purpose of informing a subsequent reimbursement decision. Federated data systems, which allow data from multiple sites to be connected for analysis, represent an opportunity to permit analysis of data from multiple jurisdictions while respecting local laws, policies, and practices. Initiatives such as the Canadian Distributed Infrastructure for Genomics platform are working towards implementation of federated data systems to enable research that could support MAAs.^189^

In sum, this approach would leverage existing organizational infrastructure but require significant and sustainable investment to support the capacity to design, negotiate, and implement MAAs. This approach has the advantage of enabling participating provinces to benefit from combined expertise and resources, while retaining the discretion to set jurisdiction-specific MAA terms. The disadvantage is the lack of uniform agreements, which creates challenges for pan-Canadian analysis, decision-making, and equitable access. If only a few provinces participate, patient accrual may be insufficient for reanalysis under an MAA. However, *ad hoc* participation initially may encourage reluctant provinces to participate once benefits are realized. Under this scenario, initial adopters bear a disproportionate burden in the initial set up, while late adopters benefit from early experience without contributing to the setup costs.

#### IV.B.2. Fully Provincial

The *status quo* approach is for individual provinces to design and implement MAAs. This approach requires the least amount of upfront administrative work and cross-jurisdictional collaboration. The provinces could cooperate to develop best practices, but ultimately, each province would design and implement a process that fits within existing administrative and data systems. Additionally, this approach would allow provinces to make decisions in the best interest of their individual populations. Data sharing and privacy concerns would be minimized, even though consent to share data with third parties may still be required by law or ethics.

However, this approach does not leverage patient data from multiple jurisdictions, which may impact analyses for rare diseases. For diseases that affect small populations, a province may have too few eligible patients to support the data collection necessary to resolve uncertainties. The individual MAA approach could also result in discordant results in different jurisdictions. Smaller provinces may also be disadvantaged due to a lack of resources and expertise to administer MAAs.

### IV.C. Policy Recommendations for MAAs in Canada

Based on the preceding review of international experience with MAAs and legal analysis, we identified twelve actionable recommendations to be considered when designing and implementing a MAA program in Canada. Our recommendations include organizational and infrastructure recommendations, such as ensuring that MAAs are supported by good governance, decision-making frameworks and processes, and fit-for-purpose data systems. Additionally, we recommend including the following best practices to ensure the appropriate use of MAAs: tailoring schemes, conducting *ex-ante* feasibility and ethical assessments, building in evaluative processes, and drafting clear withdrawal and exit plans. Lastly, we make recommendations for implementation to ensure accountability and ease transitions, including robust stakeholder engagement, considering incremental adoption, consistent and clear allocation of responsibilities, and transparency.

#### IV.C.1. Good Governance

Because of Canada’s geo-political structure, international experiences may not be transferable when developing governance structures. Supporting legal frameworks will need to align with applicable national and provincial legislation as well as the structure and function of Canada’s health care and drug funding systems. In addition, strong leadership is an essential predictor of successful MAAs.^190^ Additional human and financial resources should be provided over the medium to long term to ensure that there is sufficient capacity and expertise to adequately oversee the MAA program(s), including resources necessary to support robust governance.

Effective governance will be facilitated by clear mandates and mission statements, as well as internal administrative procedures, roles and responsibilities, funding arrangements, and goals/milestones. Depending on the governance option selected, existing organizations may need to review and update their corporate documentation and organization structures. Clear division of responsibilities and decision-making authority will be necessary to inform data collection protocols, evaluation, payments and rebates, funding, and appeals.^191^

Governance structures should also include mechanisms for representation by all relevant stakeholders, including patients, physicians, industry representatives, federal and provincial representatives (where applicable), economists, lawyers, ethicists, and methodologists. The appropriate role of manufacturers in MAAs remains unsettled and will need extra attention; for example, it may be appropriate for manufacturers to provide funding for specific activities under an MAA or participate in data sharing initiatives.^192^

#### IV.C.2. Decision-making Frameworks and Processes

Governance structures should include clear and comprehensive decision-making frameworks and processes for all decision points in the MAA process. Such frameworks can help mitigate the conflicting interests of involved stakeholders.^193^ Various decision points would require frameworks, including: when and how to initiate negotiations, determining the appropriate type of agreement, when and how to terminate or withdraw from negotiations, ensuring sufficient funding, deciding patient eligibility criteria, processes for making individual patient-level eligibility decisions, setting initial pricing, setting stop criteria, making individual patient-level stop or withdrawal decisions, termination of MAAs, data interpretation, and pricing reassessments.^194^

Some of these decisions will be more challenging than others; for example, agreeing on scheme details, such as clinical endpoints, and selecting appropriate candidate therapies for MAAs are major barriers to successful negotiation and implementation.^195^ Preconditions for when MAAs may be appropriate may include promising new treatments for unmet medical needs, comparative effectiveness uncertainties, indications with endpoints that can be easily captured in clinical practice, high-cost drugs with uncertain value, and/or indications without anticipated competition.^196^

To avoid *ad hoc* decisions and encourage transparency and certainty, conditions for decision outcomes should be pre-determined as much as is feasible, while still retaining sufficient flexibility to permit experimentation. Rather than strict criteria, a holistic checklist of factors to consider may be adopted, particularly in the early implementation phases. Existing frameworks may be useful. For example, Whittal et al. proposed a stepwise process to facilitate the negotiation process, involving: (i) assessing the product and disease profile; (ii) prioritizing evidentiary uncertainties or affordability concerns by quantifying their impact on outcomes, cost-effectiveness, cost per patient, and budget impact; (iii) identifying agreement terms that address prioritized risks; and (iv) arriving at a mutually acceptable agreement.^197^

Decision-making frameworks and processes must also include mechanisms for managing internal and external complaints and disputes. MAAs may be difficult to enforce where there is disagreement over metrics, reported outcomes, or other assessments.^198^ Experience to date with MAAs has demonstrated that disputes over decisions and outcomes can create additional uncertainty and may result in costly and lengthy dispute resolution and path dependency.^199^ One analysis found that one-third of MAAs have been subject to disputes with pharmaceutical companies or participating hospitals,^200^ which confirms the benefit of predetermined appeal and dispute resolution processes.

#### IV.C.3. Fit-For-Purpose Data Infrastructure

Appropriate health data systems are a key enabler to the successful adoption of MAAs. This includes information systems infrastructure that enables timely collection of clinical and resource use data that can address identified uncertainties.^201^ However, the extra resources, time, and costs required to collect and analyze data have been identified as a barrier to the use of MAAs.^202^ Data and evidence challenges are a leading cause of negotiation breakdown, highlighting the importance of clarifying expectations and responsibilities.^203^ Several features of data systems and considerations have been identified as critical to MAA success. First, data systems should be tailored to the governance structure and purpose of each MAA. Different approaches, including patient registries (Italy), regional databases (Spain), or integrated administrative databases (United Kingdom, Israel) have unique strengths and weaknesses that may be appropriate in different circumstances. The appropriateness of data systems should be assessed for each MAA.

Second, existing administrative health databases should be leveraged to the extent feasible. MAAs require high-quality information systems and databases and may require improved patient tracking and monitoring technologies.^204^ Where existing databases are insufficient, as is the case in much of Canada, *ad hoc* patient registries designed specifically for MAAs may be the most viable option. Third, to minimize administrative burden, data collection should be automated and integrated with existing systems where possible. Where multiple data systems are relied upon, systems should be compatible (standardized data elements) to permit meaningful analysis.^205^ Similarly, data should be reported in real-time to permit continuous and timely analysis and review.^206^

Fourth, the type and quality of data collected must be sufficient to address outstanding clinical or cost-effectiveness questions. Often, health systems do not capture the level of detail required for MAAs.^207^ The minimum data requirements should be predefined, including outcome measures that are compatible with clinical practice.^208^ The reliability of data should be considered, particularly where there is no control group.^209^ Confounding by indication, missing data, and insufficient comparable patient numbers are potential issues that could prevent meaningful data analysis.^210^ Additionally, cost and usage data may be necessary for certain types of agreements to measure performance.^211^

Fifth, defining clear roles and responsibilities of different stakeholders can help to predict positive outcomes. For example, prescribers play an important role in ensuring sufficient data quality. Low compliance from health professionals can result in low-quality and insufficient data. Incentivizing prescribers to participate in data collection could improve data quality and compliance. Additionally, engaging third parties to track and measure outcomes data may minimize interpretation disputes and facilitate trust.^212^ Funding responsibility for initial set up, ongoing maintenance, and ancillary costs associated with MAAs should be clearly and comprehensively allocated. Italy relies at least partially on industry funding for patient registries, but industry funding of data infrastructure is contentious.^213^ The extent of data sharing required and permitted under the MAA should be clearly stated at the outset of the agreement to avoid disputes.^214^ Relatedly, data ownership expectations should be predetermined. In the UK, in some cases, the data collected under an MAA is owned by the drug manufacturer; however, both approaches are contentious.^215^

#### IV.C.4. Tailored Schemes

One of the benefits of MAAs is the flexibility to use different approaches and features best suited to achieving the desired outcomes within the applicable health system. Schemes should be tailored based on the system(s) in which they will be implemented (clinical systems, health data infrastructure, payors) and the drugs and indications targeted.^216^ Features and approaches can also be mixed and matched to create hybrid agreements. Different approaches and types of MAAs introduce unique concerns, considerations, and challenges. For example, population-based agreements are often perceived as too risky by manufacturers because patient compliance and prescribing are difficult to control and track. Additionally, financial-based agreements (such as discounts) are simpler to implement, and therefore often preferred, but do not leverage post-market evidence and cannot always address uncertainties.^217^

Guiding principles emerge from experience in international jurisdictions. First, simpler models are generally preferred over complex ones. Simpler agreements are easier to implement and less costly.^218^ Second, schemes that require measuring clinical response take longer to administer than pure financial agreements and raise more administrative issues.^219^ Third, the objectives of entering into a MAA should ultimately inform the design of the MAA. Fourth, regardless of the type of MAA, clear time frames should be included in the agreement, and outcome measures should be specific.^220^ While standardized agreement length reduces administrative burden, tailoring agreement length to the uncertainties, and data needs of each agreement is preferable.^221^

Additionally, stakeholder preferences may influence the type of MAA that is implemented. Many payors prefer pay for performance (P4P) models over conditional reimbursement or CED models. P4P models typically mean that the payor only pays for patients that experience treatment benefit, and therefore, payors receive good value for their expenditure, and do not face difficult reassessments and potential delisting under conditional reimbursement or CED models.^222^

Despite the potential offered by tailored schemes, clear processes for assessing and determining the appropriate type(s) of MAAs to implement should be used to avoid *ad hoc* decisions. Each time a payor is interested in using an MAA, the benefits and challenges associated with different design types should be assessed based on the drug, indication, and stakeholders involved. Just because one type of agreement worked well in one scenario does not mean that it will work well in other situations, even if they seem comparable.^223^

#### IV.C.5. Ex-ante Feasibility Assessments

Regardless of the type of MAA that is chosen, stakeholders and governance bodies should conduct a feasibility assessment prior to implementation. For example, the appropriateness of the drug for an MAA should be assessed. MAAs may be appropriate for: high-cost drugs for priority disease areas with few or no existing effective treatments (ie. an unmet medical need); drugs which present potential long term safety or effectiveness concerns or uncertainties; drugs for which proving clinical benefit is difficult in clinical trials; drugs with potentially large but highly uncertain clinical benefit, drugs with and/or, drugs for which health effects can be determined in short time frames.^224^

Additionally, a clearly stated decision problem should be defined. Uncertainties should be clearly identified to permit the MAA to be designed with appropriate data sources and outcomes that address the uncertainties.^225^ The outcome measure(s) to be used, if applicable, and the methods for assessing outcomes should also be determined at the outset. Determining when a drug ‘works’ can be challenging, particularly in non-randomized settings. Outcomes should be objective, reproducible, clearly defined, and suitable for collection within the timeframe of the agreement. Validated biomarkers are ideal, but consideration should be given to including patient reported outcomes, where feasible. The timeframe should be considered; longer timeframes can pose additional adherence issues or changes in clinical practice. However, timeframes should be designed considering patient recruitment and the necessary time to conduct reliable clinical assessments.^226^ The evidentiary justification for entering into an MAA rather than making a conventional reimbursement decision should be established prior to drafting and entering into an agreement (ie uncertainty can be reduced through an MAA).^227^

Various pragmatic factors should also be assessed for feasibility. For example, financial feasibility, including potential budget impact, cost of executing and managing the agreement, resource demands, staffing costs, and how payments will be calculated and made, if applicable.^228^ Calculating and processing refunds can be administratively complex, and should be the responsibility of stakeholders incentivized to adhere to payment schedules.^229^ Additionally, MAAs should only be used where the benefit of additional evidence outweighs the cost of negotiating and executing the agreement.^230^ The burden that the MAA will impose on health and administrative systems, patients, physicians, and other stakeholders should also be factored into the assessment. Reliance on stakeholders, especially health care workers and administrative staff, can be a significant deterrent. Incentives for health care professionals to participate should be considered. Willingness of involved stakeholders, including patients, physicians and other healthcare professionals, and drug manufacturers to take on responsibility for various roles and responsibilities.^231^

The ability to implement the MAA within existing clinical care pathways is an additional consideration.^232^ Logistics of and responsibility for data collection necessary to address uncertainties, either through existing databases or creating and implementing a new one.^233^ This may include systems feasibility, particularly where there are multiple health systems involved, which can complicate implementation.^234^

More broadly, ex-ante feasibility assessments should include a legal assessment, including data sharing and transfer, liability, and intellectual property rights.^235^ A feasibility assessment could also include an analysis of entering into, or not entering into, the MAA.^236^ For example, certain types of MAAs may have unintended consequences on price variability and net monetary benefit.^237^ Lastly, lack of expertise and knowledge necessary to successfully negotiate, design, and implement MAAs is a major concern in most jurisdictions.^238^ As a result, dedicating resources to develop and foster the necessary expertise for MAAs is an important predictor of success. A variety of disciplinary expertise is needed to support MAAs, including operational and analytic data professionals, appropriately trained professionals to evaluate schemes, negotiation processes and management, and methodological expertise to design and studies and define outcome measures.^239^

Feasibility assessments help identify circumstances in which MAAs may not be appropriate. For example, MAAs may not be appropriate where: effective treatments already exist with proven long-term outcomes; health authorities bear the burden of disproportionate development costs; patient compliance is likely to be a challenge that has not been mitigated; the potential gain is outweighed by the administrative and financial burden; or the scheme is unlikely to produce robust evidence. *A priori* assessments can also help insulate against claims of procedural unfairness if a drug is not selected to be funded under an MAA; they demonstrate that the decision was based on predetermined criteria.^240^

#### IV.C.6. Ethical Assessment

Though ethical assessment should be integrated with ex-ante feasibility assessments and ex-post evaluation processes, we include it as a distinct recommendation because it is often overlooked in MAA implementation. MAAs have the potential to promote equitable access to novel health technologies but also have the potential to exacerbate existing ethical concerns common to clinical practice and research, as well as introduce novel ones.^241^ To protect patients and the health care system, comprehensive ethical assessments should be included in MAA governance structures, as well as MAA design and implementation processes, to identify potential ethical concerns and implement mitigation strategies, where possible. At the governance level, an ethics panel, group, or committee could be included to: oversee the overall function of the MAA program; assess any ethical issues that might arise out of decision-making, such as a decision not to pursue an MAA or to terminate a MAA; and, oversee overarching ethical analyses related to resource allocation, patient access, evidence generation, and the role of health care providers and patients. An ethics committee could also bear responsibility for monitoring the disclosure of any conflicts of interest.^242^

Individual MAAs should also be assessed for potential ethics concerns. At the feasibility stage, MAAs should be assessed to ensure that they can be ethically justified, factoring in the potential risks and benefits to patients, as well as potential impacts on the health care system. At this stage, an ethical assessment can contribute to the decision-making process on whether an MAA is appropriate. During the design and negotiation phases, potential methodological issues in the design of data collection should be screened for, including the potential for bias and recruitment challenges. Additionally, great consideration should be given to consent mechanisms and disclosures. MAAs occur at the intersection of research and care, and thus challenge legislative, regulatory, and policy requirements for obtaining informed consent.^243^ Greater consensus is needed on this topic. Nevertheless, consent processes and requirements should be thoughtfully designed based on interdisciplinary expertise and patient feedback.

Existing ethical frameworks could be leveraged and applied to MAAs, such as Accountability for Reasonableness (AFR). AFR provides a framework for decision-makers to promote substantive and procedural fairness and legitimacy when considering competing values in priority setting. AFR has four conditions to quality a process as fair and legitimate: relevance; publicity; revisability; and enforcement.^244^ Though AFR has been subject to some criticism^245^, the takeaway is that there are existing frameworks that can be used in the context of MAAs, at the very least as a starting point.

#### IV.C.7. Evaluative Processes

Lack of formal evaluation of MAAs is a significant barrier to tracking the performance of existing MAAs and improving their design and implementation.^246^ Evaluation is important throughout the term of the agreement and following its completion. Assessment should be ongoing, at predetermined intervals to ensure accurate monitoring of outcomes, including patient recruitment, data collection, costs or any other relevant variables to permit adjustments or amendments and minimize burden on patients and systems.^247^ MAA evaluations should be multidimensional, including organizational and economic impact, effectiveness at reducing uncertainty, drug utilization, and impact on industry innovation.^248^

Though little experience to date exists to inform evaluation processes and procedures, experts have shared various tips and opinions on how to best utilize evaluations for MAAs. For example, evaluations should be internal to the responsible organization, or contracted out to a trusted third party under a confidentiality agreement, to allow for evaluation of identifiable information, while protecting patient privacy. Despite this, transparency of evaluation results should be permitted to the extent possible under data sharing agreements and commercial confidentiality requirements, to promote accountability and trust among stakeholders.^249^ Striking the right balance between competing needs is an ongoing challenge and will need to be considered.

Evaluation is also important on a broader scale to track and identify and potential unintentional effects that MAAs could have on the broader health system. For example, MAAs may serve as extensions of industry marketing activities or manufacturers may overprice medicines in anticipation of rebates or other price reductions.^250^ Furthermore, the value or benefits that MAAs can offer may not be realized in practice. It is often unclear whether they are impactful enough to affect overall spending, or whether there are enough eligible products to impact spending and justify implementation and administrative costs.^251^ As a result, evaluations should be conducted to determine whether MAAs have an overall positive impact on overall spending, the patients who need access to treatments, and innovation.^252^

#### IV.C.8. Clear Withdrawal and Exit Strategies

The main concern of payors is that temporary decisions tend to become permanent, and therefore, MAAs introduce the risk that access and price will remain constant even if contrary evidence becomes available.^253^ Because of the complexity associated with temporary coverage decisions and pushback from pharmaceutical companies and patients, payors are skeptical that delisting will be a feasible option.^254^ However, payors need to be confident that reimbursement can be withdrawn or altered when results indicate that doing so is warranted. Similarly, patients have expressed apprehension that a treatment may be withdrawn from them individually, if they fail to meet response threshold, or entirely, even if they have a positive response, because the overall evidence does not support continued funding.^255^ Despite the recognized importance of clear withdrawal processes, one review of CED schemes for medical devices found that most schemes had no ex-ante decision rule linking the results to future decisions.^256^

Thoughtful contract drafting is one way to mitigate this concern by including clear processes for withdrawal or alteration of reimbursement. Decision rules, reimbursement decisions following completion of evidence generation, and withdrawal protocols should be clearly and comprehensively considered. These must be predetermined to avoid conflicts or reimbursing ineffective drugs due to inertia.^257^ External factors that could impact the agreement should also be considered, such as the launch of new drugs or changes in clinical guidelines.^258^ Particular attention should be paid if the decision is reversed to the fate of patients who have already received treatment and may be benefitting. Controlled withdrawal programs should be determined prior to the implementation of the agreement and clearly communicated to patients. Stakeholders may also wish to grandfather in patients who accessed the drug during the MAA.

#### IV.C.9. Stakeholder Engagement

Stakeholder willingness to modernize and adapt to new processes and misaligned priorities pose a barrier to adopting MAAs in Canada, and lack of stakeholder buy-in may prevent or limit the success of the MAA.^259^ As a result, early and ongoing stakeholder engagement is necessary to design and implement successful and realistic MAAs. Relevant stakeholders should be involved in all stages of the process, from design to evaluation, including patients, clinicians, manufacturers, and payors.^260^ However, it may be necessary to limit industry involvement in particular stages of the process to preserve the independence of the scheme.^261^

Despite early and ongoing engagement, obtaining stakeholder agreement on key issues is a significant challenge due to competing interests, and may result in unsuccessful negotiations or disputes.^262^ Lack of trust between stakeholders can compromise decision-making, but trust can be encouraged through increased engagement and communication.^263^ One recommendation is to map all potential results of evidence generation or reassessments included in MAAs and define the price and coverage consequences.^264^ The importance of multi-stakeholder engagement was demonstrated in the UK’s agreement to fund elosulfase alfa to treat mucopolysaccharidosis type IVA. Here, the networks developed by physicians and patient groups enabled information to be communicated to eligible patients. Additionally, patients were prioritized as a key contributor to data collection and patient assessment criteria.^265^

#### IV.C.10. Incremental Implementation

Spain’s experience of incremental adoption may be advisable in Canada to permit an adaptative approach that will allow for experimentation and adjustment. Pilot projects could facilitate early adoption to identify challenges and inefficiencies and test different solutions. However, the experience gained from pilot projects might not be transferrable to other institutions or other health systems due to different legislative and administrative constraints. Pilot projects may also be advisable at later stages in the MAA adoption process to test new features. Incremental implementation would also permit specific stakeholder engagement, such as the clinicians and administrators, which may promote buy-in and adherence.

#### IV.C.11. Consistent and Clear Funding and Resource Allocation

Securing funding to cover the added expenses associated with MAAs and investing in necessary infrastructure is consistently cited as a significant barrier to the sustainability of MAAs.^266^ MAAs are burdensome to implement and manage, requiring significant financial and administrative resources.^267^ Specifically, the cost of data collection can be substantial.^268^ Additionally, MAAs can result in additional costs beyond just the drug that is the subject of the agreement, such as hospitalizations and treatment for adverse events. It is critical to allocate responsibility for funding for the necessary administrative and data infrastructure, as well as any downstream clinical expenses, however, opinions differ on who should be responsible for additional costs.^269^ One review of CED for medical devices found that the additional costs associated with research were either covered by public funds or were subsidized (partially or fully) by application fees paid by the manufacturer.^270^ Drug manufacturers can also contribute by financing data infrastructure (such as registries) or sharing implementation costs.^271^ If manufacturers contribute financially, particularly to data collection and/or analysis efforts, conflicts of interest and control over data need to be managed.^272^

The extra costs to design and implement MAAs can be substantial. It is important to also consider costs incurred throughout the health system. For example, MAAs do not always provide funding to institutions to cover administrative and clinical resources required to collect and process patient data and financial claims, which can undermine stakeholder buy-in and compromise the integrity of the agreement. However funding responsibility is allocated, it should ensure sustainability of the agreement over time, including necessary infrastructure and human resources costs.^273^

#### IV.C.12. Transparency

Lack of transparency of existing MAAs has hampered identification of best practices. As a result, there have been numerous calls to increase transparency around MAAs. First, the objectives of the agreement should be transparently stated at the outset, so that the performance of the agreement can be easily monitored.^274^ Second, transparency around data collection and analyses is also important to permit accountability and ensure all stakeholders can review and validate the data. Of course, sensitive patient information needs to be protected, so clinical data should only be transparent to the extent that patient privacy can be respected. Controversy exists over how much information should be shared; often, MAAs will include non-disclosure clauses and commercial information is often excluded from access to information requirements.^275^ Furthermore, Quebec protects listing agreements from disclosure, permitting only the name of the drug manufacturer, the name of the medication, and the total sum received pursuant to the listing agreement to be published in annual financial reports.^276^ Third, funding arrangements should be transparent to mitigate conflicts of interest and promote accountability. Increased transparency will also have broader implications; it will permit cross-jurisdictional sharing and learning and increase collaboration. Specifically, transparency around pricing (initial pricing and after reassessment) is important, because confidential pricing can impact other countries that rely on external reference pricing.^277^

## V. CONCLUSION

Interest in alternative reimbursement models, such as MAAs, has grown significant over last past few decades as drug prices have grown, putting strain on the sustainability of health systems. While many countries to date have implemented MAAs in varying forms, Canada has only limited experience using this novel contractual tool. In this paper, we explored legal, policy, and practical challenges, considerations, and implications to adopting MAAs in Canada. One of the major challenges is determining the governance scale for implementing such agreements. While there are many benefits to a pan-Canadian approach, including efficiency, consistency, and leveraging buying power, Canada’s constitutional framework and geo-political realities pose significant barriers to adopting such an approach. Considering this, we conclude that a quasi-federal or fully provincial approach is the most realistic governance approach, despite the inherent challenges they introduce including uneven adoption and difficulties leveraging Canada’s data pool. Regardless of the approach adopted, there are several best practices and recommendations that can be extracted from international experience, academic literature, and domestic legal analysis to address or mitigate identified challenges and barriers. Strong governance systems and decision-making frameworks, fit for purpose data systems, ex-ante feasibility assessments, ethical assessments, evaluative processes, clear withdrawal and exit strategies, stakeholder engagement, incremental adoption, consistent and clear funding and resource allocation, and enhanced transparency measures have been identified as key considerations when designing and implementing MAAs to promote appropriate and efficient use of MAAs.

## Footnotes

1 Patented Medicine Prices Review Board, *CompassRx*, 7^th^ ed.*, Annual Public Drug Plan Expenditure Report, 2019/20*, https://www.canada.ca/en/patented-medicine-prices-review/services/npduis/analytical-studies/compassrx-7th-edition.html.

2 See eg, Canadian Agency for Drugs and Technology in Health, *Drugs for Rare Diseases: A Review of National and International Health Technology Assessment Agencies and Public Payers’ Decision-Making Processes*, https://www.cadth.ca/drugs-rare-diseases-review-national-and-international-health-technology-assessment-agencies-and (accessed May 26, 2020).

3 Joel Lexchin, *Formulary Status of Drugs in Ontario After Health Canada has Issued a Serious Safety Warning: A Cohort Study*, 27 JRS 135 (2015) (finding that the reimbursement status of 14/15 drugs remained unchanged after the release of a safety warning).

4 Provinces (Alberta, BC, Manitoba, New Brunswick, Newfoundland, NWT, Nova Scotia, Nunavut, Ontario, PEI, Saskatchewan, Yukon), Federal (Veterans Affairs, RCMP, Non-Insured Health Benefits, National Defense, Correctional Services Canada, Citizenship and Immigration).

5 See eg, Pharmaceutical Services Act, S.B.C. 2012, c 22 (Can.) at s 4; Ontario Drug Benefits Act, R.S.O. 1990, c O.10 (Can.) at s 19–20.

6 Canadian Agency for Drugs and Technology in Health, *Coverage Categories at Public Drugs Plans in Canada*, https://www.cadth.ca/coverage-categories-public-drugs-plans-canada (accessed July 13, 2022).

7 See eg, Gérard de Pouvourville, *Risk-Sharing Agreements for Innovative Drugs: A New Solution to Old Problems?*, 7 Eur. J. Health Econ. 155 (2006); Adrian Towse & Louis P. Garrison, *Canʼt Get No Satisfaction? Will Pay for Performance Help?: Toward an Economic Framework for Understanding Performance-Based Risk-Sharing Agreements for Innovative Medical Products*, 28 PharmacoEconomics 93 (2010); Jakub Adamski et al., *Risk Sharing Arrangements for Pharmaceuticals: Potential Considerations and Recommendations for European Payers*, 10 BMC Health Serv. Res. 153 (2010); Thomas Morel et al., *Reconciling Uncertainty of Costs and Outcomes with the Need for Access to Orphan Medicinal Products: A Comparative Study of Managed Entry Agreements across Seven European Countries*, 8 Orphanet. J. Rare Dis. 198 (2013).

8 Peter J. Neumann et al., *Risk-Sharing Arrangements That Link Payment For Drugs To Health Outcomes Are Proving Hard To Implement*, 30 Health Affairs 2329 (2011); Panos Kanavos et al., *Managing Risk and Uncertainty in Health Technology Introduction: The Role of Managed Entry Agreements*, 8 Glob. Policy 84 (2017).

9 Jesper Jørgensen & Panos Kefalas, *Annuity Payments can Increase Patient Access to Innovative Cell and Gene Therapies under England’s Net Budget Impact Test*, 5 Journal of Market Access & Health Policy 1355203 (2017); Richard Edlin et al., *Sharing Risk between Payer and Provider by Leasing Health Technologies: An Affordable and Effective Reimbursement Strategy for Innovative Technologies?*, 17 Value in Health 438 (2014); Jakub P. Hlávka et al., *The Economics of Alternative Payment Models for Pharmaceuticals*, 22 Eur. J. Health Econ. 559 (2021); Elisabeth Mahase, *UK Launches Subscription Style Model for Antibiotics to Encourage New Development*, BMJ m2468 (2020) (Also known as the Netflix model or annuity payments, leasing agreements have been used to reimburse expensive technologies, treatments that are administered only once, such as gene therapies, and public health treatments such as antibiotics and hepatitis C treatment).

10 Neumann et al., *supra* note 8; Adamski et al., *supra* note 7; Claudio Jommi, *Managed Entry Agreements and High Cost Medicines (European Perspective)*, Equitable Access to High-Cost Pharmaceuticals 35 (2018).

11 Neumann et al., *supra* note 8; Josh J. Carlson et al., *Linking Payment to Health Outcomes: A Taxonomy and Examination of Performance-Based Reimbursement Schemes between Healthcare Payers and Manufacturers*, 96 Health Policy 179 (2010).

12 Adamski et al., *supra* note 7; Jommi, *supra* note 10.

13 Jommi, *supra* note 10; Carlson et al., *supra* note 11.

14 Gregory S. Zaric & Bernie J. O’Brien, *Analysis of a Pharmaceutical Risk Sharing Agreement based on the Purchaser’s Total Budget*, 14 Health Econ. 793 (2005).

15 Jacoline C. Bouvy, Claudine Sapede & Sarah Garner, *Managed Entry Agreements for Pharmaceuticals in the Context of Adaptive Pathways in Europe*, 9 Front. Pharmacol. 280 (2018).

16 Mélanie Bourassa Forcier & François Noël, *Product Listing Agreements (PLAs): A New Tool for Reaching Quebec’s Pharmaceutical Policy Objectives?*, 9 Healthc Policy 65 (2013).

17
*Id.*; Carlo Federici et al., *Coverage with Evidence Development Schemes for Medical Devices in Europe: Characteristics and Challenges*, 22 Eur. J. Health Econ. 1253 (2021); Paul Trueman, David L. Grainger & Kristen E. Downs, *Coverage with Evidence Development: Applications and issues*, 26 Int. J. Technol. Assess Health Care 79 (2010); Simon Walker et al., *Coverage with Evidence Development, Only in Research, Risk Sharing, or Patient Access Scheme? A Framework for Coverage Decisions*, 15 Value in Health 570 (2012).

18 Josh J. Carlson et al., *Current Status and Trends in Performance-Based Risk-Sharing Arrangements Between Healthcare Payers and Medical Product Manufacturers*, 12 Appl. Health Econ. Health Policy 231 (2014).

19 Carlson et al., *supra* note 11.

20
*Id.*; Alex Bastian, Deeksha Dua & Sahand Mirzahossein, *The Use of Risk-Sharing Agreements to Manage Costs, Mitigate Risk, and Improve Value for Pharmaceutical Products*, 2 J. C. Pathw. 43 (2016).

21 In 2010, when founded the pCPA was called the pan-Canadian Pricing Alliance. In 2015 the name was changed to the pan-Canadian Pharmaceutical Alliance.

22 Participating jurisdictions include: British Columbia, Alberta, Saskatchewan, Manitoba, Ontario, Quebec, New Brunswick, Nova Scotia, PEI, Newfoundland and Labrador, Yukon, Northwest Territories, Nunavut, Non-Insured Health Benefits, Correctional Services of Canada, and Veterans Affairs Canada.

23 pan-Canadian Pharmaceutical Alliance, *pCPA Brand Process Guidelines*, (2019), https://www.pcpacanada.ca/sites/default/files/aoda/pCPA_Brand_Process_Guidelines_EN_FINAL-s.pdf (accessed July 14, 2022).

24
*Id*.

25 Forcier & Noël, *supra* note 16.

26 Steven G. Morgan et al., *Use of Product Listing Agreements by Canadian Provincial Drug Benefit Plans*, 8 Healthc Policy 45 (2013).

27 Cancer Care Ontario, *EBP Herceptin Update*, https://www.cancercareontario.ca/sites/ccocancercare/files/EBP_HerceptinRpt_Patients.pdf (accessed July 24, 2022); Cancer Care Ontario, *Evidence Building Program Policy*, https://www.cancercareontario.ca/sites/ccocancercare/files/assets/CCOEvidenceBuildingPolicy.pdf (accessed July 24, 2022) (A two-year update for Herceptin was published, which predicted that sufficient information would be available by 2016 to inform a permanent funding decision, but at the time of writing in 2022, no update had been provided).

28 Compilation of Québec Laws and Regulations, C.Q.L.R., c A-29.01, r. 2.

29 Paige A. Thomson & University of Victoria, *Negotiated Contracts for Funding Pharmaceuticals: A National Survey of Canadian Public Drug Payers*.

30
Mondher Toumi & Szymon Jaroslawski, Managed entry agreements and funding for expensive therapies 96 (2022).

31 Adamski et al., *supra* note 7.

32 Toumi & Jaroslawski, *supra* note 30.

33
*The National Institute for Health and Care Excellence (Constitution and Functions) and the Health and Social Care Information Centre (Functions) Regulations 2013*, No 259 Part 2 (2013).

34 NHS England Cancer Drugs Fund Team, *Appraisal and Funding of Cancer Drugs from July 2016 (including the new Cancer Drugs Fund): A New Deal for Patients, Taxpayers, and Industry*, https://www.england.nhs.uk/wp-content/uploads/2013/04/cdf-sop.pdf (accessed July 14, 2022).

35 National Institute for Health and Care Excellence, *Cancer Drugs Fund (2022)*, https://www.nice.org.uk/about/what-we-do/our-programmes/nice-guidance/nice-technology-appraisal-guidance/cancer-drugs-fund (accessed July 14, 2022); Centre for Health Technology Evaluation National Institute for Health and Care Excellence, *Technology Appraisals Programme: Specification for Cancer Drugs Fund Data Collection Arrangements*, https://www.nice.org.uk/Media/Default/About/what-we-do/NICE-guidance/NICE-technology-appraisal-guidance/cancer-drugs-fund/data-collection-specification.pdf (accessed July 14, 2022).

36 A. Aggarwal et al., *Do Patient Access Schemes for High-Cost Cancer Drugs Deliver Value to Society?—Lessons from the NHS Cancer Drugs Fund*, 28 Annals of Oncology 1738 (2017).

37 Nigel Hawkes, *New Cancer Drugs Fund Keeps Within £340 m a Year Budget*, BMJ k461 (2018).

38 Pharmacy and Industry Group/17080, Finance and NHS/Medicines, *The Pharmaceutical Price Regulation Scheme 2014*, https://assets.publishing.service.gov.uk/government/uploads/system/uploads/attachment_data/file/675465/The_pharmaceutical_price_regulation_scheme_2014.pdf.

39
*Id*.

40 NHS England Cancer Drugs Fund Team, *supra* note 34.

41
*Id*.

42 National Institute for Health and Care Excellence, *Guide to the Technology Appraisal and Highly Specialised Technologies Appeal Process*, https://www.nice.org.uk/process/pmg18/resources/guide-to-the-technology-appraisal-and-highly-specialised-technologies-appeal-process-pdf-72286661671621.

43 Basma v. Manchester University Hospitals NHS Foundation Trust, [2021] EWCA Civ 278.

44
*Id*.

45 Contract Variation Agreement No. 1, https://www.nice.org.uk/guidance/ta588/resources/variation-1-to-the-managed-access-agreement-pdf-9135857773 (accessed July 14, 2022).

46 Enrique Bernal-Delgado et al., *Spain: Health System Review*, 20 Health Syst. Transit. 1 (2018), Contract No.: 2.

47 Anna Reyes-Travé et al., *Characterization of the Pharmaceutical Risk-Sharing Arrangement Process in Catalonia*, 39 PharmacoEconomics 973 (2021).

48 Laura Guarga et al., *Implementing Risk-Sharing Arrangements for Innovative Medicines: The Experience in Catalonia (Spain)*, 25 Value in Health 803 (2021).

49
*Id*.

50 Ana Clopes et al., *Financial Consequences of a Payment-by-Results Scheme in Catalonia: Gefitinib in Advanced EGFR-Mutation Positive Non-Small-Cell Lung Cancer*, 20 Journal of Medical Economics 1 (2017).

51 Guarga et al., *supra* note 48.

52 Neil Grubert & MORSE, *Pharmaceutical Managed Entry Agreements: Lessons Learned from Europe, the United States, Canada and Australia*, https://morseconsulting.ca/wp-content/uploads/2018/12/International-Pharmaceutical-Managed-Entry-Agreements-2018.pdf (accessed July 14, 2022).

53 Reyes-Travé et al., *supra* note 47.

54 Italian Medicines Agency, *The Agency*, https://aifa.gov.it/l-agenzia (accessed July 14, 2022).

55 Trevor Jozef Piatkiewicz, Janine Marie Traulsen & Tove Holm-Larsen, *Risk-Sharing Agreements in the EU: A Systematic Review of Major Trends*, 2 PharmacoEconomics Open 109 (2018).

56 Grubert & MORSE, *supra* note 52.

57 Appropriateness MAAs verify use to avoid off-label use of drugs.

58 Entela Xoxi, Karen M Facey & Americo Cicchetti, *The Evolution of AIFA Registries to Support Managed Entry Agreements for Orphan Medicinal Products in Italy*, 12 Front. Pharmacol. 699466 (2021).

59 Grubert & MORSE, *supra* note 52.

60 Andrea Navarria et al., *Do the Current Performance-Based Schemes in Italy Really Work? ‘Success Fee’: A Novel Measure for Cost-Containment of Drug Expenditure*, 18 Value in Health 131 (2015).

61 Sissel Michelsen et al., *Barriers and Opportunities for Implementation of Outcome-Based Spread Payments for High-Cost, One-Shot Curative Therapies*, 11 Front. Pharmacol. 594446 (2020).

62 Navarria et al., *supra* note 60.

63 Szymon Jarosławski & Mondher Toumi, *Market Access Agreements for Pharmaceuticals in Europe: Diversity of Approaches and Underlying Concepts*, 11 BMC Health Serv. Res. 259 (2011).

64 Francisco R Gonçalves et al., *Risk-Sharing Agreements, Present and Future*, 12 ecancer (2018).

65 Xoxi, Facey & Cicchetti, *supra* note 58.

66 Neumann et al., *supra* note 8.

67
*Medicare Program: Medicare Coverage of Innovative Technolopgy (MCIT) and Definition of ‘Reasonable and Necessary’,* 86 FR 2897 (2021).

68 Centers for Medicare & Medicaid Services, *Coverage with Evidence Development*, https://www.cms.gov/Medicare/Coverage/Coverage-with-Evidence-Development (accessed July 15, 2022).

69 Annemarie Relyea-Chew, *Ethical Considerations in CMS’s Coverage With Evidence Development*, 8 Journal of the American College of Radiology 838 (2011); Sean R Tunis et al., *Improving the Quality and Efficiency of the Medicare Program Through Coverage Policy: Urban Institute* (2011); Neumann et al., *supra* note 8.

70 Centers for Medicare & Medicaid Services, *CMS Proposes Medicare Coverage Policy for Monoclonal Antibodies Directed Against Amyloid for the Treatment of Alzheimer’s Disease*, https://www.cms.gov/newsroom/press-releases/cms-proposes-medicare-coverage-policy-monoclonal-antibodies-directed-against-amyloid-treatment (accessed July 15, 2022).

71 John Wilkerson & Gabrielle Wanneh, *Though Strict, CMS Aduhelm Policy May Create Pressure for Wider Coverage*, 25 Inside Washington Publishers’ Inside CMS (2022).

72 Centers for Medicare & Medicaid Services, *Monoclonal Antibodies Directed Against Amyloid for the Treatment of Alzheimer’s Disease*, https://www.cms.gov/medicare-coverage-database/view/ncacal-decision-memo.aspx?proposed=Y&NCAId=305 (accessed July 15, 2022).

73 Shuli Brammli-Greenberg et al., *How Managed Entry Agreements Can Improve Allocation in the Public Health System: A Mechanism Design Approach*, 22 Eur. J. Health Econ. 699 (2021).

74 Christian Lovis & Ronni Gamzu, *Big Data in Israeli Healthcare: Hopes and Challenges Report of an International Workshop*, 4 Isr. J. Health Policy Res. 61 (2015).

75 Constitution Act, 1982, s 35, *being* Schedule B to the Canada Act 1982 (UK), 1982, c 11 at ss 91–92.

76 Carter v. Canada (Attorney General), 2015 S.C.C. 5, at para. 53.

77 Martha Butler & Marlisa Tiedermann, *The Federal Role in Health and Health Care*, https://lop.parl.ca/sites/PublicWebsite/default/en_CA/ResearchPublications/201191E (accessed July 15, 2022).

78 Constitution Act, 1982, s 35, *being* Schedule B to the Canada Act 1982 (UK), 1982, c 11.

79 Government of Canada & Canadian Food Inspection Agency, *Strengthening Canada’s World-Class Food Safety System Incorporation by Reference: Discussion Document,*  https://inspection.canada.ca/DAM/DAM-aboutcfia-sujetacia/STAGING/text-texte/food_actionplan_incorp_ref_discuss_1401723700118_eng.pdf (accessed July 15, 2022).

80 Colleen Flood et al., *Universal Pharmacare and Federalism*, https://irpp.org/research-studies/universal-pharmacare-and-federalism-policy-options-for-canada/ (accessed July 15, 2022).

81 Jan Kurman, *Learning from The Failures of Past Pharmacare Efforts*, https://www.ifsd.ca/en/blog/last-page-blog/past-failures (accessed July 15, 2022).

82
Halsbury’s Laws of Canada, Administrative Law (2018 Reissue), (2018), HAD-1 ‘Purposes of administrative law’.

83 Boehringer Ingelheim (Canada) v. Canadian Agency for Drugs and Technologies in Health, 2008 55998 (ON SCDC) (The issue has not yet been considered in federal court or a court in any other province, so the decision is only binding in Ontario, but it is likely to be persuasive.).

84 Canada (Minister of Citizenship and Immigration) v. Vavilov, 2019 S.C.C. 65.

85 Baker v. Canada (Minister of Citizenship and Immigration), [1999] 2 S.C.R. 817.

86 The other basis was an application to restrain publication or for damages because of the publication of misleading or inaccurate information, based on defamation, malicious falsehood, and/or negligent misrepresentation.

87 Elaine MacPhail & Barb Shea, *An Inside Look at the Early History of the CADTH Common Drug Review in Canada*, https://www.cadth.ca/sites/default/files/pdf/early_history_of_CDR.pdf (accessed July 15, 2022).

88 Health Insurance Act, R.S.O. 1990, c H.6 at s 20(1).

89 McGregor v. Alberta (Out-of-Country Health Services Appeal Panel), 2007 ABQB 138.

90 Boehringer Ingelheim (Canada) v. Canadian Agency for Drugs and Technologies in Health, 2008 55998 (ON SCDC).

91 Janssen Inc v. Minister of Health and Social Services, 2019 QCCA 39.

92 Amir Attaran, *Take Your Medicine?: The Risk of Patient-Led Legislation in Canada’s Medicine Access System*, McGill J. Law Health (2009).

93 Martineau v. Matsqui Disciplinary Board, [1980] 1 S.C.R. 602; Cardinal v. Director of Kent Institution, [1985] 2 S.C.R. 643; Knight v. Indian Head School Division No 19, [1990] 1 S.C.R. 653.

94 Attaran, *supra* note 92.

95 Sarah Burningham, *Courts, Challenges, and Cures: Legal Avenues for Patients with Rare Diseases to Challenge Health Care Coverage Decisions*, 1 Canadian Journal of Comparative and Contemporary Law 317 (2015).

96
*Id.*; Canada v. Inuit Tapirisat, [1980] 2 S.C.R. 735.

97 Burningham, *supra* note 95.

98 Hilary Young, Cuthbertson v. Rasouli*: Continued Confusion Over Consent-Based Entitlements to Life Support*, 52 ALR 745, (2015).

99 Steven G Morgan et al., *Inter-Jurisdictional Cooperation on Pharmaceutical Product Listing Agreements: Views from Canadian Provinces*, 13 BMC Health Serv. Res. 34 (2013). The free-rider problem refers to a type of market failure when those who do not pay for a service or public good, still benefit from them. In this case, provinces that opt not to enter into an MAA, and therefore avoid the costs associated with their implementation, can still benefit from the evidence generated by provinces who do enter into MAAs. Whipsawing refers to strategic price negotiations, where a manufacturer offers lower prices to early adopters to increase political pressure on other buyers.

100 Government of Canada; Indigenous Services Canada, *Jordan’s Principle*, https://www.sac-isc.gc.ca/eng/1568396042341/1568396159824 (accessed July 15, 2022).

101
*Id*.

102 Laura Glowacki, *Federal Government to Cover $2.8 m Drug for Eastern Ontario Toddler,* CBC, Jan. 18, 2021.

103 Canadian Institutes of Health Research, Natural Sciences and Engineering Research Council of Canada & Social Sciences and Humanities Research Council of Canada, *Tri-Council Policy Statement: Ethical Conduct for Research Involving Humans* (2018).

104
*Id*.

105
*Id*.

106
*Id*.

107
*Id*.

108 Emily A. Largent, Steven Joffe & Franklin G. Miller, *Can Research and Care Be Ethically Integrated?*, 41 Hastings Center Report 37 (2011).

109 Health Canada, *Building a National Strategy for Drugs for Rare Diseases: What We Heard from Canadians*, https://www.canada.ca/en/health-canada/programs/consultation-national-strategy-high-cost-drugs-rare-diseases-online-engagement/what-we-heard.html (accessed July 15, 2022).

110 Department of Finance Government of Canada, *Moving Forward on Implementing National Pharmacare*, https://www.budget.gc.ca/2019/docs/themes/pharmacare-assurance-medicaments-en.html (accessed July 15, 2022).

111 Prime Minister’s Office Government of Canada, *Delivering for Canadians Now*, https://pm.gc.ca/en/news/news-releases/2022/03/22/delivering-canadians-now (accessed July 15, 2022).

112 Canadian Agency for Drugs and Technologies in Health, *The pan-Canadian Advisory Panel on a Framework for a Prescription Drug List*, https://www.cadth.ca/pan-canadian-advisory-panel-framework-prescription-drug-list (accessed July 15, 2022).

113 Health Canada Government of Canada, *Moving Forward on a Pan-Canadian Health Data Strategy*, https://www.canada.ca/en/public-health/programs/pan-canadian-health-data-strategy.html (accessed July 15, 2022).

114 Canadian Agency for Drugs and Technologies in Health, *CADTH Post-Market Drug Evaluation Proposal Program Overview*, https://www.cadth.ca/sites/default/files/PDME/PMDE-Program-Overview-jan2022.pdf (accessed July 18, 2022).

115 Health Canada Government of Canada, *Forward Regulatory Plan: 2021–2023*, https://www.canada.ca/en/health-canada/corporate/about-health-canada/legislation-guidelines/acts-regulations/forward-regulatory-plan/plan.html (accessed July 18, 2022).

116 Food and Drugs Act, R.S.C. 1985, c F-27; Health Canada Government of Canada, *Regulatory Innovation for Health Products: Enabling Advanced Therapeutic Products*, https://www.canada.ca/en/health-canada/corporate/about-health-canada/activities-responsibilities/strategies-initiatives/health-products-food-regulatory-modernization/advanced-therapeutic-products.html (accessed July 18, 2022).

117 Bill C-11, *An Act to enact the Consumer Privacy Protection Act and the Personal Information and Data Protection Tribunal Act and to Make Consequential and Related Amendments to Other Acts*, 2nd Sess, 43rd Parl, 2020 (introduction and first reading).

118 Protecting Canadians from Unsafe Drugs Act (Vanessa’s Law), S.C. 2014, c 24.

119 Health Canada Government Of Canada, *Amendments to the Food and Drugs Act: Guide to New Authorities (Power to Require and Disclose Information, Power to Order a Label Change, Power to Order a Recall, Power to Require Assessments, and Power to Require Tests, Studies, etc.)*, https://epe.lac-bac.gc.ca/100/201/301/weekly_acquisitions_list-ef/2021/21-12/publications.gc.ca/collections/collection_2021/sc-hc/H164-307-2021-eng.pdf (accessed July 18, 2022).

120 Eldridge v. British Columbia (Attorney General), 3 S.C.R. 624, (1997).

121 Auton (guardian ad litem of) v. British Coumbia (Attorney General), 2004 S.C.C. 78.

122 Chaoulli v. Quebec (Attorney General), 2005 S.C.C. 35; Cambie Surgeries Corporation et al. v. Attorney General of British Columbia (Attorney General), 2020 BCSC 1310.

123 Gosselin v. Quebec, 2002 S.C.C. 84.

124 Enumerated grounds are those listed in section 15(1): race, national or ethnic origin, color, religion, sex, age, or mental or physical disability. Analogous grounds are those determined by the courts to be similar to enumerated grounds, including non-citizenship, marital status, sexual orientation, and aboriginality-residence.

125 R. v. Kapp, 2008 S.C.C. 41, at para. 17; Withler v. Canada (Attorney General), 2011 S.C.C. 12, at para. 30.

126 Burningham, *supra* note 95.

127 Armstrong v. British Columbia (Ministry of Health), 2010 B.C.C.A. 56.

128 Genetic Non-Discrimination Act, S.C. 2017, c 3 at ss 3, 6.

129 Louis P. Garrison et al., *A Review of Public Policy Issues in Promoting the Development and Commercialization of Pharmacogenomic Applications: Challenges and Implications*, 40 Drug Metabolism Reviews 377 (2008).

130 Constitution Act, 1982, s 35, *being* Schedule B to the Canada Act 1982 (UK), 1982, c 11.

131 Basma v. Manchester University Hospitals NHS Foundation Trust, [2021] EWCA Civ 278.

132 Food and Drug Regulations, C.R.C. c 180.

133
*Id*.

134
*Id.*; Health Canada Government of Canada, *The Preparing and Submitting Summary Reports for Marketed Drugs and Natural Health Products*—*Guidance Document for Industry*, https://www.canada.ca/en/health-canada/services/drugs-health-products/reports-publications/medeffect-canada/preparing-submitting-summary-reports-marketed-drugs-natural-health-products-guidance-industry.html (accessed July 20, 2022).

135 Protecting Canadians from Unsafe Drugs Act (Vanessa’s Law), S.C. 2014, c 24.

136 Health Canada Government of Canada, *ARCHIVED: Amendments to the Food and Drugs Act: Guide to New Authorities (Power to Require and Disclose Information, Power to Order a Label Change and Power to Order a Recall)*, https://www.canada.ca/en/health-canada/services/drugs-health-products/legislation-guidelines/amendments-food-drugs-act-guide-new-authorities-power-require-disclose-information-power-order-label-change-power-order-recall.html (accessed July 20, 2022).

137 Thomas J. Moore & Curt D. Furberg, *Electronic Health Data for Postmarket Surveillance: A Vision Not Realized*, 38 Drug Saf 601 (2015).

138 Real-World Evidence and Outcomes-Based Agreements Working Group, *2021 Research and Outputs: Executive Summary*, https://static1.squarespace.com/static/58fd16af1b631b1afffae9e0/t/61d5fb5b787eb97065ce84bf/1641413467439/2021_RWE_OBA_WorkingGroup_ExecSummary.pdf?mc_cid=674b33396a&mc_eid=948aa023e9 (accessed July 20, 2022).

139 Canadian Agency for Drugs and Technologies in Health, *CADTH Establishes New Post-Market Drug Evaluation Program*, https://www.cadth.ca/news/cadth-establishes-new-post-market-drug-evaluation-program (accessed July 20, 2022).

140 Canadian Agency for Drugs and Technologies in Health, *supra* note 114.

141 J. Stephen Mikita et al., *Determining the Suitability of Registries for Embedding Clinical Trials in the United States: A Project of the Clinical Trials Transformation Initiative*, 55 Ther. Innov. Regul. Sci 6 (2021).

142 Canada Health Act, R.S.C. 1985, c C-6.

143 Cancer Care Ontario Ontario Health, *Policy: Public Funding of Cancer Drugs within the Context of Clinical Trials*, https://www.cancercareontario.ca/sites/ccocancercare/files/assets/CCOClinicalTrialsFundingPolicy.pdf (accessed July 20, 2022).

144 Ministry of Health Government of British Columbia, *Limited Coverage Drugs*, https://www2.gov.bc.ca/gov/content/health/practitioner-professional-resources/pharmacare/pharmacies/limited-coverage-drugs (accessed July 20, 2022).

145 Telus Health, *Ontario Limited Use and Section 8*, https://assets.ctfassets.net/rz9m1rynx8pv/1hMGJPIjDQiMFCrsOzg7wo/46daebb3a5af6493537814cb2573d776/Ontario_Limited_Use_and_Section_8_2020.pdf (accessed July 20, 2022).

146 Pharmaceutical Services Act, S.B.C. 2012, c 22 at s 4; Ontario Drug Benefits Act, R.S.O. 1990, c O.10 at ss 19–20; Government of Alberta, *Drug Benefit List: Introduction*, https://idbl.ab.bluecross.ca/idbl/DBL/dbl_sec1_intro.pdf (accessed July 24, 2022).

147 Compilation of Québec Laws and Regulations, C.Q.L.R., c A-29.01, r 2.

148 Subramaniam Thanimalai, Wai Yee Choon & Kenneth Kwing-Chin Lee, *Stakeholder Views of Managed Entry Agreements: A Literature Review of National Studies*, 2 Health Policy OPEN 100032 (2021); Natalie Bohm et al., *The Challenges of Outcomes-Based Contract Implementation for Medicines in Europe*, 40 PharmacoEconomics 13 (2022).

149 Securities are any form of ownership or beneficial interest in a business entity.

150 Halsbury’s Laws of Canada, Securities (2018 Reissue) HSC-21 *Terminolog*y.

151 Halsbury’s Laws of Canada, Securities (2018 Reissue) HSC-126 *Publication of Material Change*.

152 Canadian Securities Administrators, *National Instrument 51-102*—*Continuous Disclosure Obligations, s.1.1(1)*, https://www.bcsc.bc.ca/-/media/PWS/New-Resources/Securities-Law/Instruments-and-Policies/Policy-5/51102-NI-February-9-2021.pdf (accessed July 15, 2022).

153 Halsbury’s Laws of Canada, Securities (2018 Reissue) HSC-261 *Liability for Damages*; Halsbury’s Laws of Canada, Securities (2018 Reissue) HSC-260 *Common Law Liability*.

154 Edward J. Markey, *June 1, 2005 – ‘Conspiracy of Silence: How the FDA Allows Drug Companies to Abuse the Accelerated Approval Process’*, https://www.markey.senate.gov/news/press-releases/june-1-2005-conspiracy-of-silence-how-the-fda-allows-drug-companies-to-abuse-the-accelerated-approval-process (accessed Jan 11, 2023).

155 Theratechnologies Inc v. 121851 Canada Inc, 2015 S.C.C. 18.

156 Grady v. Canada, 2008 ONCA 659; Attis v. Canada, 2008 ONCA 660.

157 Lorian Hardcastle, *Government Tort Liability for Negligence in the Health Sector: A Critique of the Canadian Jurisprudence*, 37 Queen’s law journal 525 (2012).

158 Mostly, this refers to hospitals.

159
Gerald B. Robertson & Ellen I. Picard, Legal liability of doctors and hospitals in Canada (5^th^ ed. 2017).

160 See eg, MacPhail v. Desrosiers, [1998] NSJ No 353 at para. 16 (Can.) [hospital found liable for failure to follow a policy prohibiting a woman from driving after receiving an abortion]; Comeau v. Saint John Regional Hospital, [2001] NBJ No 450 at para. 2 (Can.) [hospital liable for failure to enforce a policy requiring internists to consult with emergentologists in diagnosis and discharge]; Braun Estate v. Vaughan, [2000] MJ No 63 at para. 11 (Can.) [finding an affirmative duty to have policies for reviewing test results]; Martin v. Listowel Memorial Hospital, [1998] OJ No 3126 at para. 60 (Gen. Div.) [hospital found liable for failure to have policies or procedures for nurses to follow when admitting premature mothers].

161 Reibl v. Hughes, [1980] SCJ No 105, 114 DLR (3d) (S.C.C.).

162 Timothy Caulfield & Kerry Siminoski, *Physicians’ Liability and Drug Formulary Restrictions*, 166 CMAJ 458 (2002); Blake Murdoch & Timothy Caulfield, *The Law and Ethics of Switching from Biologic to Biosimilar in Canada*, 3 Journal of the Canadian Association of Gastroenterology 228 (2020); Law Estate v. Simice, 21 CCLT (2d) 228 (BCSC), aff’d (1995) 17 BCLR (3d) 1, 19 CCLT (2d) 127 (CA).

163 Hilary Young, *A Proposal for Access to Treatment Contrary to Clinical Judgment*, 11 McGill Journal of Law and Health 1 (2017); Hilary Young, *Why Withdrawing Life-Sustaining Treatment Should Not Require ‘Rasouli Consent’,* 6 McGill Journal of Law and Health 54 (2012); Cuthbertson v. Rasouli, 2013 S.C.C. 53; Hilary Young, Cuthbertson v. Rasouli*: Continued Confusion Over Consent-Based Entitlements to Life Support*, 52 ALR 745 (2015).

164 Lambert v. Lastoplex Chemicals Co, [1972] SCR 569 at page 574; Rivtow Marine Ltd v. Washington Iron Works, [1974] SCR 1189 at page 1200.

165 Provincial regulation of competition is usually limited to specific industries, for example, provincial monopolies on alcohol retail sales. Competition law as it pertains to pharmaceuticals is largely regulated under the Federal *Competition Act*. As a result, the scope of this document is limited to federal legislation.

166 Competition Act, R.S.C. 1985, c C-34.

167
*Id*.

168 Competition Bureau Government of Canada, *Abuse of Dominance Enforcement Guidelines*, https://www.competitionbureau.gc.ca/eic/site/cb-bc.nsf/eng/04420.html (accessed July 24, 2022).

169 Competition Act, R.S.C. 1985, c C-34.

170
*Id*.

171 Leah Z. Rand & Aaron S. Kesselheim, *Performance-Linked Reimbursement and the Uncertainty of Novel Drugs*, 15 Circ: Cardiovascular Quality and Outcomes (2022); Thanimalai, Choon & Lee, *supra* note 148; Relyea-Chew, *supra* note 69.

172 Halsbury’s Laws of Canada, Labour (2020 Resissue) HLA-463-4 *General Enforcement of Collective Agreement*.

173 Food and Drug Regulations, C.R.C. c 180.

174 Israel Ministry of Health, *Real-World Epidemiological Evidence Collaboration Agreement*, https://govextra.gov.il/media/30806/11221-moh-pfizer-collaboration-agreement-redacted.pdf (accessed July 15, 2022).

175 Canadian Intellectual Property Office Government of Canada, *Patentable Subject-Matter under the Patent Act*, https://www.ic.gc.ca/eic/site/cipointernet-internetopic.nsf/eng/wr04860.html (accessed July 24, 2022).

176 Patent Act, R.S.C. 1985, c P-4.

177 Katherine L. Bonter, Carmela De Luca & Christi J. Guerrini, *Gene Patents in Canada: Is There a New Legal Landscape?*, 22 Mol. Diagn. Ther. 149 (2018).

178 Constitution Act, 1982, s 35, *being* Schedule B to the Canada Act 1982 (UK), 1982, c 11; Colleen Flood et al., *Universal Pharmacare and Federalism*, https://irpp.org/research-studies/universal-pharmacare-and-federalism-policy-options-for-canada/ (accessed July 15, 2022); Martha Jackman, *The Constitutional Basis for Federal Regulation of Health*, 5 health law review 3 (1996).

179 References re Greenhouse Gas Pollution Pricing Act, 2021 S.C.C. 11 [hereinafter *Reference re GGPPA*].

180 RJR-Macdonald v. Canada (Attorney General), [1995] 3 SCR 199 at para. 200; R v. Swain, [1991] 1 SCR 933.

181 R v. Wetmore, [1983] 2 S.C.R. 284.

182 Reference re Assisted Human Reproduction Act, 2010 S.C.C. 61.

183 Nova Scotia v. Attorney General of Canada, [1951] S.C.R. 31.

184 Flood et al., *supra* note 80.

185 Department of Finance Government of Canada, *supra* note 110.

186 pCPA, *Personal Communication*.

187 Melissa Thompson et al., *Targeting Improved Patient Outcomes using Innovative Product Listing Agreements: A Survey of Canadian and International Key Opinion Leaders*, 8 CEOR 427 (2016).

188 Personal Health Information Protection Act, 2004, SO 2004, c. 3.

189 L. Jonathan Dursi et al., *CanDIG: Federated Network across Canada for Multi-Omic and Health Data Discovery and Analysis*, 1 Cell Genomics 100033 (2021).

190 Clopes et al., *supra* note 50.

191 Bohm et al., *supra* note 148.

192 Thompson et al., *supra* note 187.

193 Bouvy, Sapede & Garner, *supra* note 15.

194 Grubert & MORSE, *supra* note 52; Bohm et al., *supra* note 148; Kanavos et al., *supra* note 8.

195 Federici et al., *supra* note 17; Trevor Jozef Piatkiewicz, Janine Marie Traulsen & Tove Holm-Larsen, *Risk-Sharing Agreements in the EU: A Systematic Review of Major Trends*, 2 PharmacoEconomics Open 109 (2018); Amanda Whittal et al., *Facilitating More Efficient Negotiations for Innovative Therapies: A Value-Based Negotiation Framework*, 38 Int J Technol Assess Health Care e23 (2022).

196 Hans-Georg Eichler et al., *Exploring the Opportunities for Alignment of Regulatory Postauthorization Requirements and Data Required for Performance-Based Managed Entry Agreements*, 37 Int J Technol Assess Health Care e83 (2021).

197 Whittal et al., *supra* note 195.

198 Rand & Kesselheim, *supra* note 171.

199 Basma v. Manchester University Hospitals NHS Foundation Trust, [2021] EWCA Civ 278.

200 Katelijne van de Vooren et al., *Market-Access Agreements for Anti-Cancer Drugs*, 108 J. R. Soc. Med. 166 (2015).

201 Kanavos et al., *supra* note 8; Thanimalai, Choon & Lee, *supra* note 148; Adamski et al., *supra* note 7.

202 Real-World Evidence and Outcomes-Based Agreements Working Group, *Outcomes-Based Agreements: Canadian Experience and Perceptions*, http://static1.squarespace.com/static/58fd16af1b631b1afffae9e0/t/618931aef49c9002c7e7e59a/1636381102563/RWE+OBA+Working+Group_Canadian+OBA+Survey+Results_Oct+12+2021.pdf?mc_cid=6bcafb9a31&mc_eid=948aa023e9 (accessed July 24, 2022).

203 Nirosha Mahendraratnam et al., *Value-Based Arrangements may be more Prevalent than Assumed*, 25 Am. J. Manag. Care 70 (2019).

204 Piatkiewicz, Traulsen & Holm-Larsen, *supra* note 195; Neumann et al., *supra* note 8.

205 Neumann et al., *supra* note 8; Joshua D. Wallach et al., *Feasibility of Using Real-world Data to Emulate Postapproval Confirmatory Clinical Trials of Therapeutic Agents Granted US Food and Drug Administration Accelerated Approval*, 4 JAMA Netw. Open e2133667 (2021).

206 Carlos Gustavo García-Collado et al., *Impact of a Risk-Sharing Agreement in Rheumatoid Arthritis in Spain*, 125 Health Policy 335 (2021); Thanimalai, Choon & Lee, *supra* note 148.

207 Neumann et al., *supra* note 8.

208 Thanimalai, Choon & Lee, *supra* note 148; Bouvy, Sapede & Garner, *supra* note 15.

209 Adamski et al., *supra* note 7.

210 Bouvy, Sapede & Garner, *supra* note 15.

211 Joseph A. Goble et al., *Performance-Based Risk-Sharing Arrangements: U.S. Payer Experience*, 23 J Manag. Care Spec. Pharm. 1042 (2017).

212 Bouvy, Sapede & Garner, *supra* note 15; Bohm et al., *supra* note 148; Real-World Evidence and Outcomes-Based Agreements Working Group, *supra* note 202.

213 Thompson et al., *supra* note 187; Federici et al., *supra* note 17; Adamski et al., *supra* note 7.

214 Thanimalai, Choon & Lee, *supra* note 148.

215 Thompson et al., *supra* note 187; Federici et al., *supra* note 17; Adamski et al., *supra* note 7.

216 Rick A Vreman et al., *Application of Managed Entry Agreements for Innovative Therapies in Different Settings and Combinations: A Feasibility Analysis*, 17 IJERPH 8309 (2020).

217 Thanimalai, Choon & Lee, *supra* note 148.

218 Whittal et al., *supra* note 195; Bouvy, Sapede & Garner, *supra* note 15.

219 van de Vooren et al., *supra* note 200.

220 Devidas Menon et al., *Principles of Design of Access with Evidence Development Approaches: A Consensus Statement from the Banff Summit*, 28 PharmacoEconomics 109 (2010).

221 Michael Drummond et al., *Coverage with Evidence Development for Medical Devices in Europe: Can Practice Meet Theory?*, Health Economics hec.4478 (2022).

222 Bouvy, Sapede & Garner, *supra* note 15.

223 Kanavos et al., *supra* note 8.

224 Toumi & Jaroslawski, *supra* note 30; Adamski et al., *supra* note 7; Thompson et al., *supra* note 187; Alexander T. Sandhu et al., *Perks and Pitfalls of Performance-Linked Reimbursement for Novel Drugs: The Case of Sacubitril-Valsartan*, 15 Circ: Cardiovascular Quality and Outcomes (2022).

225 Bob Stevens et al., *Elosulfase alfa in the Treatment of Mucopolysaccharidosis Type Iva: Insights from the First Managed Access Agreement*, 16 Orphanet. J. Rare Dis. 394 (2021); Whittal et al., *supra* note 195; Martin Wenzl & Suzannah J. Chapman, *Performance-Based Managed Entry Agreements for New Medicines in OECD Countries and EU Member States*, 115 OECD Working Papers (2019).

226 Robert W. Dubois, Kimberly Westrich & Lisabeth Buelt, *Are Value-Based Arrangements the Answer We’ve Been Waiting for?*, 23 Value in Health 418 (2020); Jesper Jørgensen & Panos Kefalas, *The Use of Innovative Payment Mechanisms for Gene Therapies in Europe and the USA*, 16 Regen. Med. 405 (2021); Louis P. Garrison et al., *Performance-Based Risk-Sharing Arrangements—Good Practices for Design, Implementation, and Evaluation: Report of the ISPOR Good Practices for Performance-Based Risk-Sharing Arrangements Task Force*, 16 Value in Health 703 (2013); Whittal et al., *supra* note 195; Thompson et al., *supra* note 187; Grubert & MORSE, *supra* note 52; Neumann et al., *supra* note 8; Carlson et al., *supra* note 11.

227 Adamski et al., *supra* note 7; Walker et al., *supra* note 17; Thompson et al., *supra* note 187.

228 Garrison et al., *supra* note 226; Whittal et al., *supra* note 195; Thompson et al., *supra* note 187; Thanimalai, Choon & Lee, *supra* note 148; Bouvy, Sapede & Garner, *supra* note 15.

229 Bohm et al., *supra* note 148.

230 Wenzl & Chapman, *supra* note 225.

231 Eichler et al., *supra* note 196; van de Vooren et al., *supra* note 200.

232 Thanimalai, Choon & Lee, *supra* note 148; Stevens et al., *supra* note 225; Neumann et al., *supra* note 8; Adamski et al., *supra* note 7.

233 Stevens et al., *supra* note 225; Thompson et al., *supra* note 187; Whittal et al., *supra* note 195; Jørgensen & Kefalas, *supra* note 226; Garrison et al., *supra* note 226.

234 Thanimalai, Choon & Lee, *supra* note 148.

235 Eichler et al., *supra* note 196.

236 García-Collado et al., *supra* note 206.

237 Zaric & O’Brien, *supra* note 14.

238 Thanimalai, Choon & Lee, *supra* note 148; Real-World Evidence and Outcomes-Based Agreements Working Group, *supra* note 202.

239 Mahendraratnam et al., *supra* note 203; Bohm et al., *supra* note 148; Grubert & MORSE, *supra* note 52; Leslie Levin et al., *Coverage with Evidence Development: The Ontario Experience*, 27 Int. J. Technol. Assess Health Care 159 (2011); Federici et al., *supra* note 17; Adamski et al., *supra* note 7.

240 Adamski et al., *supra* note 7.

241 Jan R.R. Lewis, Ian Kerridge & Wendy Lipworth, *Coverage With Evidence Development and Managed Entry in the Funding of Personalized Medicine: Practical and Ethical Challenges for Oncology*, 33 JCO 4112 (2015).

242 Adamski et al., *supra* note 7; *Id*.

243 Lewis, Kerridge & Lipworth, *supra* note 241.

244 Mireille Goetghebeur et al., *The Art of Priority Setting*, 389 The Lancet 2368 (2017); Monika Wagner et al., *Moving Towards Accountability for Reasonableness—A Systematic Exploration of the Features of Legitimate Healthcare Coverage Decision-Making Processes Using Rare Diseases and Regenerative Therapies as a Case Study*, 8 Int. J. Health Policy Manag. 424 (2019); Norman Daniels & James Sabin, *Limits to Health Care: Fair Procedures, Democratic Deliberation, and the Legitimacy Problem for Insurers*, 26 Philosophy & Public Affairs 303 (1997).

245 Katharina Kieslich & Peter Littlejohns, *Does Accountability for Reasonableness Work? A Protocol for a Mixed Methods Study using an Audit Tool to Evaluate the Decision-Making of Clinical Commissioning Groups in England: Table 1*, 5 BMJ Open e007908 (2015); Sigurd Lauridsen & Kasper Lippert-Rasmussen, *Legitimate Allocation of Public Healthcare: Beyond Accountability for Reasonableness*, 2 Public Health Ethics 59 (2009).

246 Stevens et al., *supra* note 225; Gonçalves et al., *supra* note 64.

247 Stevens et al., *supra* note 225; Adamski et al., *supra* note 7.

248 Jommi, *supra* note 10.

249 Thanimalai, Choon & Lee, *supra* note 148.

250 Bohm et al., *supra* note 148.

251 Dubois, Westrich & Buelt, *supra* note 226.

252 Menon et al., *supra* note 220.

253 Bohm et al., *supra* note 148.

254 Lewis, Kerridge & Lipworth, *supra* note 241; Neumann et al., *supra* note 8; Adamski et al., *supra* note 7.

255 Thanimalai, Choon & Lee, *supra* note 148; Sarah Costa et al., *Public Perspectives on Disinvestments in Drug Funding: Results from a Canadian Deliberative Public Engagement Event on Cancer Drugs*, 19 BMC Public Health 977 (2019).

256 Federici et al., *supra* note 17 (The Netherlands provides an exception; the level of effectiveness that must be demonstrated during the scheme to obtain unconditional reimbursement is predefined at the onset).

257
*Id.*; Adamski et al., *supra* note 7; Garrison et al., *supra* note 226.

258 Jommi, *supra* note 10.

259 Carlson et al., *supra* note 11; Real-World Evidence and Outcomes-Based Agreements Working Group, *supra* note 202; Mahendraratnam et al., *supra* note 203.

260 Thompson et al., *supra* note 187.

261 Menon et al., *supra* note 220.

262 Federici et al., *supra* note 17.

263 Real-World Evidence and Outcomes-Based Agreements Working Group, *supra* note 202; Thanimalai, Choon & Lee, *supra* note 148.

264 Bouvy, Sapede & Garner, *supra* note 15.

265 Stevens et al., *supra* note 225.

266 Toumi & Jaroslawski, *supra* note 30; Federici et al., *supra* note 17; García-Collado et al., *supra* note 206.

267 Grubert & MORSE, *supra* note 52.

268 Bohm et al., *supra* note 148.

269 Kanavos et al., *supra* note 8.

270 Federici et al., *supra* note 17.

271 Thanimalai, Choon & Lee, *supra* note 148.

272 Neumann et al., *supra* note 8.

273
*Id.*; Adamski et al., *supra* note 7.

274 Menon et al., *supra* note 220; Wenzl & Chapman, *supra* note 225.

275 Pierluigi Russo et al., *Medicine Price Transparency and Confidential Managed-Entry Agreements in Europe: Findings from the Euripid Survey*, 125 Health Policy 1140 (2021).

276 Regulation Respecting the Conditions Governing the Accreditation of Manufacturers and Wholesalers of Medications, C.Q.L.R., c A-29.01, r 2.

277 Mahendraratnam et al., *supra* note 203; Kanavos et al., *supra* note 8; Thanimalai, Choon & Lee, *supra* note 148.

